# Small Extracellular Vesicles Promote Axon Outgrowth by Engaging the Wnt-Planar Cell Polarity Pathway

**DOI:** 10.3390/cells14010056

**Published:** 2025-01-06

**Authors:** Samar Ahmad, Tania Christova, Melanie Pye, Masahiro Narimatsu, Siyuan Song, Jeffrey L. Wrana, Liliana Attisano

**Affiliations:** 1 Department of Biochemistry, Donnelly Centre, University of Toronto, Toronto, ON M5S 3E1, Canada; samar.ahmad@mail.utoronto.ca (S.A.); tania.christova@utoronto.ca (T.C.); siyuan.song@utoronto.ca (S.S.); 2 Center for Systems Biology, Lunenfeld-Tanenbaum Research Institute, Mt. Sinai Hospital, Toronto, ON M5G 1X5, Canada; pye@lunenfeld.ca (M.P.); narimatsu@lunenfeld.ca (M.N.); wrana@lunenfeld.ca (J.L.W.); 3 Department of Molecular Genetics, University of Toronto, Toronto, ON M5G 1X5, Canada

**Keywords:** extracellular vesicles, axon outgrowth, planar cell polarity, neurite outgrowth, cortical neurons

## Abstract

In neurons, the acquisition of a polarized morphology is achieved upon the outgrowth of a single axon from one of several neurites. Small extracellular vesicles (sEVs), such as exosomes, from diverse sources are known to promote neurite outgrowth and thus may have therapeutic potential. However, the effect of fibroblast-derived exosomes on axon elongation in neurons of the central nervous system under growth-permissive conditions remains unclear. Here, we show that fibroblast-derived sEVs promote axon outgrowth and a polarized neuronal morphology in mouse primary embryonic cortical neurons. Mechanistically, we demonstrate that the sEV-induced increase in axon outgrowth requires endogenous Wnts and core PCP components including Prickle, Vangl, Frizzled, and Dishevelled. We demonstrate that sEVs are internalized by neurons, colocalize with Wnt7b, and induce relocalization of Vangl2 to the distal axon during axon outgrowth. In contrast, sEVs derived from neurons or astrocytes do not promote axon outgrowth, while sEVs from activated astrocytes inhibit elongation. Thus, our data reveal that fibroblast-derived sEVs promote axon elongation through the Wnt-PCP pathway in a manner that is dependent on endogenous Wnts.

## 1. Introduction

Neurons are the fundamental unit of the central nervous system (CNS) and exhibit a polarized morphology comprised of a single long axon and one or multiple dendrites. Sensory signals are received by dendrites and propagated down the length of the axon to neighboring neurons [1,2,3,4]. Thus, proper neuronal morphology is critical for synaptic transmission and cognitive functions of the brain. To acquire a polarized morphology, one neurite outgrows the other minor neurites and forms an axon, a process referred to as neuronal polarization [1,2,3,4]. Axons differ in their growth potential such that during development axons grow rapidly, but in the adult CNS, axons have lost the ability to grow [5]. Hence, the study of the mechanisms of axon outgrowth during development can provide insights into processes that need to be reactivated during axon regeneration in adults. Extracellular factors including Wnts, which are known to associate with extracellular vesicles (EVs), have been reported to modulate neurite outgrowth and neuronal morphology [1,2,3,4].

Exosomes are small extracellular vesicles (sEVs) that arise from multivesicular bodies (MVBs) within the endosomal pathway. Exosomes range in size from 30–150 nm and harbor a variety of biomolecules, including proteins, DNA, and RNAs [6,7,8]. Exosomes regulate many processes including immune responses, wound healing, and disease pathology [9,10,11]. In the CNS, exosomes act as carriers for communication among neurons and glia and promote neuroprotective functions [12,13,14,15,16,17]. The biological activities of exosomes depend upon the features of the cells from which they originate. For instance, exosomes from mesenchymal stem cells increase the number of neurites and promote neurite outgrowth in cortical neurons [18,19,20], while those from Schwann cells of the peripheral nervous system (PNS) promote axon regeneration in PNS neurons [21]. Peripheral nerve fibroblast-derived exosomes promote Schwann cell-mediated myelination of neurons [22]. Moreover, fibroblasts promote sorting of Schwann cells at an injury site that leads to axon regrowth [23]. Thus, both fibroblasts and Schwann cells contribute to the remarkable regenerative ability of the PNS. In the context of growth-inhibitory substrates, it has been shown that exosomes from fibroblasts can promote axon regeneration in cortical neurons [24], suggesting that exosomes may have therapeutic potential. However, the direct effect of fibroblast-derived exosomes on axon outgrowth of CNS neurons during growth-permissive conditions remains unclear. We previously showed that exosomes from fibroblasts can promote cell protrusion and motility in cancer cells [25]. Given that core mechanisms of cytoskeletal remodeling are conserved during non-neuronal cell migration and neurite outgrowth in neurons, we sought to examine whether exosomes might similarly play a role in modulating neurite outgrowth. Thus, the current study is aimed at investigating the axon outgrowth-promoting properties of fibroblast-derived exosomes.

Exosomes have previously been shown to mobilize autocrine Wnt-planar cell polarity (PCP) signaling in breast cancer cells [25]. PCP signaling was first described for its function in modulating tissue polarity by aligning cells in a single plane, perpendicular to the apical-basal axis [26,27,28,29]. In vertebrates, PCP signaling also functions in the development of the inner ear, orientation of hair follicles, and convergent extension (CE) movements during gastrulation and neural tube closure [30,31,32,33,34,35]. In the CNS, PCP modulates numerous developmental processes including differentiation of neural progenitor cells, neuronal migration, axon guidance, and dendritic arborization [31,36,37,38]. Core components of the PCP pathway are the seven-pass transmembrane Frizzled (Fzd) receptors, four-pass transmembrane Van-Gogh-like (Vangl) proteins, and cytoplasmic proteins including Prickle (Pk) and Dishevelled (Dvl) [26,27,28,29]. Unlike in Drosophila, Wnt morphogens play an important role in regulating PCP signaling in vertebrates and may act as an instructive cue [33].

Wnts are lipid-modified secreted glycoproteins that act as morphogens to regulate processes during embryonic development, including cell proliferation and fate, polarity, and migration [39,40,41,42,43]. In neurons, Wnts are known to modulate dendritogenesis, axon guidance and remodeling, synaptogenesis, synaptic plasticity, and differentiation [44,45]. In mammals, there are 19 Wnts that bind to different classes of cell surface receptors and trigger diverse cellular responses [41]. Wnt receptors and co-receptors include members of Fzd, low-density lipoprotein related receptor (LRP), and receptor tyrosine kinases (RTKs) such as ROR, RYK, and PTK7 [46,47]. Despite increasing knowledge about Wnt signaling, little is known about the role of the Wnt-PCP pathway in axon outgrowth.

The current study was aimed at investigating the contribution of Wnt-PCP signaling in exosome-induced axon outgrowth. We show that sEVs derived from diverse fibroblast cell lines promote axon outgrowth that leads to acquisition of a polarized neuronal morphology. Mechanistically, we show that the sEV-induced increase in axon outgrowth requires neuronally expressed Wnts and PCP components including Pk, Vangl, Fzd, and Dvl. Importantly, we show that sEVs are internalized by neurons, where they can colocalize with Wnt7b and also induce a shift in Vangl2 localization towards the distal part of the axon. In contrast to fibroblast-derived sEVs, those isolated from neurons or astrocytes do not alter axon outgrowth, while sEVs isolated from activated astrocytes inhibit axon outgrowth. Overall, the current study uncovers the potential of fibroblast-derived sEVs in activating axon outgrowth systems through PCP signaling and may also indicate the therapeutic potential of sEVs in treating neuronal injury.

## 2. Materials and Methods

### 2.1. Mice

Mouse studies were conducted in accordance with the guidelines of the Canadian Council on Animal Care (CCAC) and approved by the University Animal Care Committee (UACC), University of Toronto or The Centre for Phenogenomics (TCP), Toronto, Ontario, Canada. Mice were housed in individually ventilated cages with free access to standard animal food and water in a climate-controlled room with a 12 h light/dark cycle. CD1 pregnant females E15-16 were purchased from Charles River.

*Prickle1 (Pk1)* conditional knockout mice were generated by introducing the *Pk1* targeting vector from the European Mouse Mutagenesis Consortium (EUCOMM) Program, HTGR03009_Z_6_E08) into G4 embryonic stem (ES) cells by electroporation. ES cell colonies were selected using G418 (Invitrogen, Waltham, MA, USA, 10131027), and genomic DNA was analyzed by Southern blotting to identify homologous recombinants. A targeted *Pk2* ES cell clone was from the EUCOMM Program (HEPD0638_2_E01 clone C3). Chimeric embryos were produced at the Transgenic Core of the TCP facility by diploid aggregation of targeted ES cells and CD1 embryos.. Chimeric mice were crossed with ICR mice, obtained from TCP, to obtain the F1 heterozygotes, which were then crossed with Flp recombinase mice to remove the neomycin selection cassette. For the *Pk1* allele, genomic DNA was digested with NdeI and probed with a *Pk1* 3′ probe, resulting in a 15.7 kb (wild-type allele) and a 10.4 kb (mutant allele) fragment. For the *Pk2* allele, genomic DNA was digested with BstEII, ScaI, NsiI, AvrII, or Bsu36 and probed with a Neo probe to produce targeted bands of 15 kb, 18.5 kb, 17.6 kb, 9.5 kb or 12.7 kb, respectively. Single floxed mice (*Pk1*^fl/fl^; *Pk2^+/+^* and *Pk1^+/+^*; *Pk2^fl/fl^*) were crossed with each other to obtain *Pk1/2* double floxed mice (*Pk1^fl/fl^*; *Pk2^fl/fl^*). To obtain the brain specific conditional knockout, single floxed mice and double floxed mice were crossed with a *Nestin-Cre* line, acquired from TCP, to obtain *Cre+ (Pk1^fl/fl^*; *Pk2^+/+^*; *Cre+*, *Pk1^+/+^*; *Pk2^fl/fl^*; *Cre+*, *Pk1^fl/fl^*; *Pk2^fl/fl^*; *Cre+*) and *Cre*− (*Pk1^fl/fl^*; *Pk2^+/+^*; *Cre*−, *Pk1^+/+^*; *Pk2^fl/fl^*; *Cre*−, *Pk1^fl/fl^*; *Pk2^fl/fl^*; *Cre*−) floxed mice, which were subsequently crossed with each other to obtain the various genotype combinations. For genotyping, genomic DNA from a 2 mm E16.5 tail piece was isolated and subjected to PCR using primers listed in Table 1. Loss of Pk1/2 expression was confirmed in brain sections from *Pk1^fl/fl^*; *Pk2^fl/fl^*; *Nestin-Cre+* mice by immunostaining with Pk1/2 antibodies (Table 2), followed by confocal microscopy.

Heterozygous *Vangl2^Lp/+^* (*Vangl2^S464N^*) mice [48], obtained from Jackson Labs (Ellsworth, ME, USA, #000220), were kindly provided by Dr. Helen McNeil (Lunenfeld-Tanenbaum Research Institute, Mount Sinai Hospital, Toronto, ON, Canada) and crossed with each other to obtain homozygous mutants (*Vangl2^Lp/Lp^*). *Smurf1/2* heterozygous mice (*Smurf1^+/−^; Smurf2^+/−^*) were generated previously [49] and crossed with each other to generate single knockout (*Smurf1^−/−^* or *Smurf2^−/−^*) embryos.

**Table 2:** List of antibodies.

Antibodies	Source	Identifier
Mouse anti-MAP-2 (Clone AP20, 1:500)	Millipore (Darmstadt, Germany)	MAB3418
Mouse anti-Tubulin β 3 (Clone Tuj1, 1:3000)	Biolegend (San Diego, CA, USA)	801202
Mouse anti-Tau-1 (Clone PC1C6, 1:300)	Millipore	MAB3420
Mouse anti-TSG101 (1:1500)	GeneTex (Irvine, CA, USA)	GTX70255
Mouse anti-Flotillin-1 (1:1500)	BD Transduction (Franklin Lakes, NJ, USA)	618021
Mouse anti-CD81 (B-11, 1:1500)	Santa Cruz (Dallas, TX, USA)	sc-166029
Mouse anti-Prickle1 (F-5, 1:100)	Santa Cruz	Sc-393034
Mouse anti-Prickle2 (Clone 3B4.1, 1:100)	Millipore	MABN1529
Rabbit anti-GFAP (1:500)	Dako Agilent (Santa Clara, CA, USA)	Z0334
Rabbit anti-GFP (1:400)	Invitrogen	A11122
Rabbit anti-β III Tubulin (1:500)	Abcam	ab18207
Rabbit anti-Calnexin (1:2000)	[50]	N/A
Rat anti-Vangl2 (Clone 2G4, 1:200)	Millipore	MABN750
Rat anti-HA (1:1000)	Roche Canada (Mississauga, ON, Canada)	11867423001
Goat anti-Wnt7b (1:200)	R&D Systems (Minneapolis, MN, USA)	AF3460
Goat anti-Wnt5a (1:1000)	R&D Systems	AF645
Goat anti-GFP (1:1000)	Rockland Immunochemicals (Limerick, PA, USA)	600-101-215
Donkey anti-goat DyLight^TM^ 488 (1:1000)	Thermo Fisher Scientific	SA5-10086
Donkey anti-goat Alexa Fluor^TM^ 647 (1:1000)	Thermo Fisher Scientific	A21447
Goat anti-Mouse Alexa Fluor^®^ 488 (1:1000)	Invitrogen	A11029
Goat anti-Rabbit Alexa Fluor^®^ 488 (1:1000)	Invitrogen	A11034
Goat anti-Mouse Alexa Fluor^TM^ 488 (1:500)	Thermo Fisher Scientific	A21121
Goat anti-Rat Alexa Fluor^TM^ 488 (1:1000)	Thermo Fisher Scientific	A11006
Goat anti-Mouse Alexa Fluor^TM^ 568 (1:500)	Thermo Fisher Scientific	A21134
Goat anti-Mouse Alexa Fluor^®^ 594 (1:1000)	Invitrogen	A11032
Goat anti-Rabbit Alexa Fluor^®^ 594 (1:1000)	Invitrogen	A11012
Goat anti-rabbit Alexa Fluor^TM^ 647 (1:1000)	Thermo Fisher Scientific	A21244
Donkey anti-Goat IgG-HRP (1:5000)	Jackson ImmunoResearch (West Grove, PA, USA)	705-035-147
Donkey anti-Mouse IgG-HRP (1:5000)	Jackson ImmunoResearch	715-035-150
Donkey anti-Rabbit IgG-HRP (1:5000)	Jackson ImmunoResearch	711-035-152
IgG F2.A	[51]	N/A
IgG	[52]	N/A

### 2.2. Isolation, Culturing, and Transfection of Mouse Cortical Neurons

Primary cortical neurons were isolated from mouse embryos E15.5-16.5 as previously described [53]. Briefly, brains were harvested and placed in ice-cold HBSS (Gibco, Waltham, MA, USA), and cerebral cortices were dissected. Cortices were digested in 0.25% trypsin-EDTA (Gibco, 25200056) at 37 °C, followed by trypsin inactivation by adding Dulbecco’s Modified Eagle Medium (DMEM) (Gibco, 11995-065) containing 10% FBS (Gibco, 12483-020). After further mechanical dissociation, neurons were seeded at a density of 30,000 cells/well for regular experiments or 125,000 cells/well for transfection experiments in 4-well chamber slides (Lab-Tek II, Waltham, MA, USA, 155382) coated with poly-L-lysine (20 μg/mL, Sigma, Saint Louis, MI, USA, P1399) and laminin (2 μg/mL, Corning, New York, NY, USA, 354232). Neurons were cultured at 37 °C and 5% CO_2_ in Neurobasal medium (Gibco, 21103-049) supplemented with 2% B-27 (Gibco, 17504-044), 0.5% N-2 (Gibco, 17502-048), 2 mM GlutaMAX^TM^ (Gibco, 35050-061), and 0.5% penicillin/streptomycin (Gibco, 15140-122).

For transfections, dissociated neurons were electroporated with cDNA (2 μg) or siRNA (Table 3) using an Amaxa nucleofector (program-005, Lonza, Basel, Switzerland) and mouse neuron nucleofector^TM^ kit (VPG-1001, Lonza) according to the manufacturer’s instructions. Briefly, neurons (4–5 × 10^6^) were electroporated with a mix of 2 μM siGENOME SMARTpool siRNAs (Dharmacon, Cambridge, UK), comprised of a pool of four individual siRNAs except for the siWnt7a pool, which contained two individual siRNAs (Table 3), and 2 μg of enhanced green fluorescent protein (eGFP) as a transfection reporter. Media were replaced 4 h after seeding. All DNAs were prepared using an Endotoxin-Free Maxi-prep kit (Qiagen, Hilden, Germany).

### 2.3. Mammalian Cell Culture and Transfection

L cells (CRL-2648), human dermal fibroblasts (HDFn) (PCS-201-010™), BJ cells (CRL-2522^TM^), and MDA-MB-231 cells were purchased from ATCC (Manassas, VA, USA); normal human lung fibroblasts (NHLF) were purchased from Lonza (CC-2512); and mouse embryonic fibroblasts (MEFs) were acquired from the TCP facility. Mouse L cells stably expressing Wnt5a (L-Wnt5a) were generated earlier as reported previously [54] and cultured in DMEM containing 10% FBS and 0.4 mg/mL G418 (Thermo Fisher Scientific, Waltham, MA, USA 10131035). MDA-MB-231 cells were cultured in Roswell Park Memorial Institute (RPMI) 1640 medium containing 5% FBS, while all other lines were cultured in DMEM containing 10% FBS. Cell lines were routinely tested for mycoplasma contamination using MycoAlert^TM^ Plus reagent (Lonza, LT07-703).

For siRNA transfections, L cells (1 × 10^6^) were transfected with 40 nM siGENOME SMARTpool siRNAs (Dharmacon) using Lipofectamine^TM^ RNAiMAX reagent (Invitrogen, 13778075) according to the manufacturer’s protocols. For cDNA transfection of MDA-MB-231 cells, plasmids (2 μg) were delivered using Lipofectamine^®^ 3000 (Invitrogen, L3000-001) according to the manufacturer’s instructions. The Wnt7b construct was generated using the Gateway system (Life Technologies, Carlsbad, CA, USA) by transfer from an entry clone into a C-terminal HA-tagged pCMV5-based destination vector.

### 2.4. Primary Astrocyte Cultures

Dissociated primary astrocytes were isolated from the cortices of CD1 mouse post-natal (P1–P4) pups as previously published [55]. Dissociated cells were cultured in DMEM supplemented with heat-inactivated 10% FBS and 1% penicillin/streptomycin in 75 cm^2^ flasks (Corning, 430720U) coated with poly-D-lysine (50 μg/mL, Sigma, P7280). Media were changed after 24 h of initial seeding, and every 2 days thereafter. Cultures were grown for 8 days and then shaken at 120 rpm overnight to remove microglia and less adherent cells. Cells were then washed, trypsinized, and these astrocyte-enriched cultures were used for subsequent experiments.

For 3D cultures, astrocytes were seeded in a gel containing type-I rat tail collagen (Ibidi, Gräfelfing, Germany, 50201), prepared according to the manufacturer’s protocol. Briefly, 10% minimum essential media (MEM, Gibco, 11430-030), 1% GlutaMAX^TM^, and collagen (2 mg/mL) were mixed on ice and neutralized using 1 M NaOH. Subsequently, DMEM containing cells was gently added to the gel mixture, resulting in a cell density of 2 × 10^6^ cells/mL of gel mixture. The gel mixture was transferred to either 6-well plates (2.5 mL/well) for sEV isolation or chamber slides (700 μL/well) for immunofluorescence microscopy. The liquid gel mixture was placed at 37 °C and 5% CO_2_ for 30 min to solidify, and then fresh DMEM containing heat-inactivated 10% FBS and 1% penicillin/streptomycin was added.

### 2.5. Preparation of Conditioned Media

Fibroblasts (0.5 × 10^6^) were seeded in a 100 mm dish and grown to confluency. Cells were washed with DMEM and incubated with DMEM either without FBS (L cells and L-Wnt5a) or exosome-free 10% FBS (for NHLF, HDFn, BJ, MEFs) (Gibco). After 3–4 days, conditioned media (CM) was collected, centrifuged at 300× *g* to remove cells and stored at 4 °C. CM from neurons was prepared from primary cortical neurons (4 × 10^6^ cells) seeded in a 100 mm dish and cultured in serum-free complete medium. On day 4, 25% of freshly made complete medium supplemented with cytosine β-d-arabinofuranoside (Sigma, Saint Louis, MI, USA, C1768) (1.25 μM final concentration) was added, and CM was collected on day 8. For 2D astrocytes, enriched primary astrocyte cells were seeded in p75 flasks and cultured in DMEM supplemented with heat-inactivated exosome-free 10% FBS. Cultures were grown to confluency, and the CM was collected after 6 days. For 3D astrocyte cultures in collagen gel, enriched primary astrocytes were seeded in 6-well plates, and CM was collected after 6 days. Small EVs from fibroblast-CM were purified within 1 week, whereas sEVs from neurons and astrocytes were isolated on the same day.

### 2.6. Isolation of Small Extracellular Vesicles (sEVs)

Conditioned media (CM) (50 mL) was collected from fibroblasts, neurons, or astrocytes and centrifuged at 300× *g* for 5 min and at 2000× *g* for 20 min to remove cells and large debris, respectively. Supernatants were then centrifuged at 10,000× *g* for 30 min to remove apoptotic bodies and large vesicles. The resulting supernatants were filtered through a 0.2 μm filter (Thermo Fisher Scientific, 568-0020) and ultracentrifuged at 100,000× *g* (Type 70 Ti rotor, Beckman Coulter, Brea, CA, USA) for 2 h using 25 × 89 mm polycarbonate tubes (Beckman Coulter, 355618). Pellets were resuspended in 500 μL of sterile phosphate-buffered saline (PBS) and diluted with 7.5 mL of PBS. Diluted pellets were ultracentrifuged at 100,000× *g* (Type 70.1 Ti rotor, Beckman Coulter) for 2 h using 16 × 76 mm polycarbonate tubes (Beckman Coulter, 355603). The resulting pellets were resuspended in 250 μL of sterile PBS, aliquoted, and stored at −80 °C.

### 2.7. Purification of sEVs Using Iodixanol Density Gradient

A discontinuous iodixanol gradient, prepared by diluting 60% iodixanol (Optiprep^TM^, Sigma, D1556) with 0.25 M sucrose/10 mM Tris, pH 7.4, was used to further purify the sEV pellets (100,000× *g*). The sEV pellet, resuspended in 500 μL of PBS, was overlaid on top of the discontinuous gradient (40%, 20%, 10%, and 5%; 3 mL each, but 2.5 mL for 5%) and ultracentrifuged at 100,000× *g* (SW 40 Ti or SW41 Ti rotor, Beckman Coulter) for 16 h using ultra-clear tubes (Beckman Coulter, 344060 or 344059). Twelve fractions of 1 mL each were collected manually from the top. Fractions were washed by diluting with 7 mL of PBS and ultracentrifuged at 100,000× *g* (Type 70.1 Ti rotor, Beckman Coulter) for 2 h. The resulting pellets were resuspended in 150 μL of sterile PBS and stored at −80 °C.

### 2.8. Purification of sEVs by Size Exclusion Chromatography

The sEV pellets (100,000× *g*) were purified using the qEV original size exclusion column with a pore size of 70 nm (Izon science, Christchurch, New Zealand, 1001683) according to the manufacturer’s protocol with minor modifications. Briefly, the column was equilibrated with 30 mL of PBS, and the sEV pellet, resuspended in 500 μL of PBS, was overlaid on the qEV column, followed by elution with PBS. Fractions of 500 μL were collected and analyzed for particle and protein concentration using NTA and the Bradford assay, respectively.

### 2.9. Purification of sEVs by Exo-spin^TM^ Precipitation

The sEVs from 3D-astrocytes were purified using the Exo-spin^TM^ purification kit (Cell Guidance Systems, Cambridge, UK) according to the manufacturer’s instructions. Briefly, CM was precleared by sequential centrifugations at 300× *g*, 2000× *g* and 10,000× *g*. Supernatants were mixed with the precipitation buffer and incubated overnight at 4 °C. Samples were centrifuged at 10,000× *g* for 30 min, and the pellets were resuspended in 200 μL of PBS. Samples were further purified using the provided size exclusion columns, and sEVs were eluted in 200 μL of PBS.

### 2.10. Proteinase K and Detergent Treatment of sEVs

sEVs were incubated with a final concentration of 100 μg/mL Proteinase K or 1% Triton X-100 at 37 °C for 1 h with gentle vortexing every 15 min. After treatment, sEVs were washed in 7 mL of PBS, ultracentrifuged for 2 h, and the resulting pellet was resuspended in 120 μL of PBS.

### 2.11. Nanoparticle Tracking Analysis

The number of particles was measured using the ZetaView^®^ PMX 110 device (Particle Metrix GmbH, Inning am Ammersee, Germany). The measurements were taken at all 11 different positions, with camera sensitivity set to 90 and shutter speed at 45 using the ZetaView software (version 8.03.08). The video quality was set to medium, and the data were analyzed using the ZetaView analysis software (version 8.02.31) with the following post-acquisition parameters: minimum size 10, maximum size 1000, and minimum brightness 20.

### 2.12. Small EV Internalization Assay

For sEV internalization assays, 50 mL of media was collected from L cells stably transfected with either CD81-EYFP or CD81-pLVX as a control [25]. CM was centrifuged at 300× *g* for 5 min and at 2000× *g* for 20 min to remove cells and large debris, respectively. Supernatants were concentrated 10-fold using a 10 kDa MWCO filter (Amicon^®^ Ultra-15, Merck Millipore, Darmstadt, Germany, UFC901008). Neurons were seeded in 8-well Ibidi chambers and treated with 10X CM for 29 h or 30 min, prior to fixation, immunostaining, and imaging using confocal microscopy.

### 2.13. Neurite Outgrowth Assay in a Microfluidic Chamber

Two-compartment microfluidic chambers containing a 150 μm microgroove were purchased from Xona Microfluidics (Durham, NC, USA, XC150). Chambers were prepared for seeding neurons according to the manufacturer’s instructions with a few modifications. Briefly, chambers were treated with a pre-coat solution and subsequently coated with poly-L-lysine (20 μg/mL) and laminin (2 μg/mL). Cortical neurons (50,000 cells) were seeded in one well in an initial volume of 20 μL and allowed to adhere for 5 min at 37 °C and 5% CO_2_. Complete Neurobasal medium was added to all the wells, and neurons were cultured for 5 days until axons crossed the microgroove and emerged in the distal chamber. The sEVs (5 μg/mL) were added either to the somal side or axonal side on day 5 for 24 h prior to fixation and immunostaining.

### 2.14. FACS Cell Sorting

Neurons (3–4 × 10^6^) electroporated with siRNA and GFP were seeded in a 6-well plate. Media were replaced 4 h after plating, and cells were trypsinized at 33 h. The cell suspension was centrifuged, and the pellet was resuspended in 300 μL of FACS buffer (1X PBS containing 1 mM EDTA, 25 mM HEPES and 1% Bovine Serum Albumin (BSA), pH 7.4). The cell suspension was filtered and then subjected to GFP-based sorting using a BD Influx™ cell sorter (University of Toronto, Flow Cytometry Facility). GFP-positive and negative cell populations were isolated with the following instrument settings: pressure: 12 psi, nozzle size: 100 μm. RNA was isolated from GFP-positive cells and qPCR was performed as described in Section 2.15.

### 2.15. RT and qPCR

For neurons and astrocytes, total RNA was extracted using the Norgen Single-Cell RNA Purification Kit (Norgen, Thorold, ON, Canada, 51800). For other cells, total RNA was extracted using the PureLink RNA Mini Kit (Life Technologies, 12183025). Complementary DNA was synthesized using 35 to 200 ng of purified RNA, using oligo-dT primers and M-MLV Reverse Transcriptase (Invitrogen, 28025-013). Real-time qPCR was performed using SYBR^TM^ Green PCR Master Mix (Applied Biosciences, Salt Lake City, UT, USA, 4309155) on the QuantStudio 6 Flex System (Applied Biosciences). Relative gene expression was quantified using the ΔΔCt method and normalized to glyceraldehyde-3-phosphate dehydrogenase (Gapdh). Primers used are listed in Table 4.

### 2.16. Immunofluorescence Microscopy and Immunoblotting

For immunocytochemistry, neurons and astrocytes were fixed in 4% paraformaldehyde as previously described [53]. Briefly, cells were permeabilized with 0.2% Triton/PBS, washed with PBS, and blocked at room temperature for 1 h with 3% BSA/PBS. Cells were incubated with primary antibodies for 2 h at room temperature or overnight at 4 °C, washed three times with PBS, and incubated with secondary antibodies for 2 h at room temperature. Cells were washed with PBS and stored in PBS or in Ibidi mounting medium prior to imaging. For immunohistochemistry of mouse embryos at E15.5, embryos were fixed in 4% paraformaldehyde in PBS at 4 °C overnight. After washing with 0.1% Tween 20 in PBS (PBST) three times for 5 min, the samples were cryoprotected by immersion in 15% sucrose in PBS at 4 °C overnight, then in 30% sucrose in PBS at 4 °C overnight. Samples were embedded in optimum cutting temperature (OCT) compound, and cryosections of 16 μm were prepared with a cryostat (Leica Biosystems, Nussloch, Germany, CM3050 S) and then stained with antibodies listed in Table 2. For epifluorescence microscopy, images were acquired using the 40X Plan-NEOFLUAR objective of a Zeiss Axiovert 200 M epifluorescence microscope. For confocal microscopy, images were acquired using a Zeiss Axio Observer Z1 microscope equipped with Zen Blue software (version 2, Zeiss, Oberkochen, Germany), a spinning disc confocal scanner (CSU-X1, Yokogawa, Japan), and a CCD camera (Axiocam 506 mono) with the following objectives: Plan-Apochromat 20x/0.8 M27 air, Plan-Apochromat 40x/1.4 Oil DIC, and Plan-Apochromat 63x/1.4 Oil DIC M27. Some confocal images were acquired using a Nikon ECLIPSE Ti2 inverted microscope equipped with a LUA-S laser unit and NIS-Elements software version 5.41.00 (from Nikon Canada Inc., Mississauga, ON, Canada,) with the following objectives: Nikon CFI Apo-LWD 40X/1.15 WI and CFI Apo-LWD 20X/0.95 WI. All images were acquired in a random fashion.

For immunoblotting, cells were lysed in lysis buffer (50 mM Tris-HCl, 150 mM NaCl, 1 mM EDTA, 0.5% Triton X-100, 1 mM DTT containing protease and phosphatase inhibitors) [56]. Protein concentration was determined using Bradford reagent (BIORAD, Hercules, CA, USA). Proteins from cell lysates (1–20 μg) and sEVs (1–10 μg) were separated on an SDS-PAGE gel and analyzed by immunoblotting as described previously [57].

### 2.17. Electron Microscopy

sEVs were visualized using a Talos L120C transmission electron microscope (TEM) (Thermo Scientific) in the Microscopy Imaging Laboratory, University of Toronto, Canada. The sEV samples were frozen on TEM grids (Quantifoil, Groblöbichau, Germany, R2,2 300 mesh, EMS) that were charged for 30 s with a Pelco EasiGlow glow discharge cleaning system (Ted Pella, Redding, CA, USA). Samples (4 μL) were deposited onto the grid, which was then blotted and plunge frozen using a Vitrobot IV (FEI). Images were taken at 120 kV with magnifications of 28,000X and 57,000X, corresponding to a pixel size of 510 and 249 pm, respectively. Images were collected at a defocus of −4 to −1.5 microns using the TEM Image and Analysis (TIA) software (version 5.0 SP4, Thermo Fisher Scientific).

### 2.18. Quantification

For Tuj1 stained neurons, the length of all neurites (including the longest neurite, a prospective axon, and dendrites, all other neurites excluding the longest neurite) was quantified using Volocity software version 6.5.1 (Quorum Technologies Inc., Puslinch, ON, Canada), as reported previously [53,54]. In MAP2 and Tau-1 experiments, neurons were classified into two groups: only MAP2-positive minor neurites or minor neurites and a single Tau-1-positive axon displaying medial-to-distal localization of Tau-1.

For Vangl2 localization, the intensity of Vangl2 in the soma, proximal axon, and distal axon was quantified using Nikon NIS-Elements software (Version 5.41.00). For sEVs and Wnt7b co-localization, neurons were identified using Tuj1 as a reference channel, and the Pearson’s colocalization coefficient for GFP (sEVs) and Wnt7b was determined using Nikon NIS-Elements software (Version 5.41.00).

### 2.19. Statistical Analyses

The data for axon length and longest neurite length are plotted as violin plots from 90–120 neurons per condition in three independent experiments. The data were analyzed statistically using an unpaired *t*-test for comparing two groups, one-way ANOVA with Dunnett’s post-test for multiple comparisons to a single control group, or two-way ANOVA with Tukey’s post-test for multiple comparisons to control and between samples, as indicated in the figure legends. All statistics were done in GraphPad PRISM 9 (GraphPad software Inc., La Jolla, CA, USA). Statistical significance: * *p* < 0.05, ** *p* < 0.01, *** *p* < 0.001.

## 3. Results

### 3.1. sEVs Promote Acquisition of a Polarized Neuronal Morphology by Enhancing Axon Outgrowth

**Figure 1:**
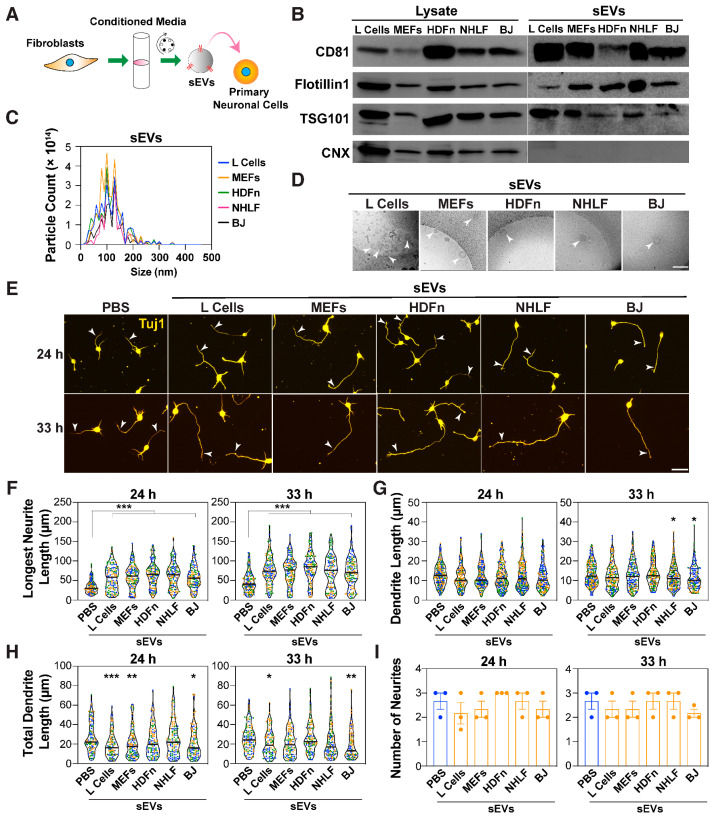
sEVs promote the growth of the prospective axon. (**A**) A schematic of the experimental set up. Mouse cortical neurons (E15.5-16.5) are treated with sEVs isolated from fibroblast-conditioned media (CM) using differential centrifugation. (**B**) Representative immunoblotting of cell lysates and sEV pellets (100,000× *g*) from the indicated fibroblast cell lines for EV markers CD81, Flotillin1, and TSG101, and the ER protein, calnexin (CNX). (**C**) Nanoparticle tracking analysis (NTA) of differential centrifugation pellets. A representative plot indicating the particle size distribution from three independent purifications is shown. (**D**) Representative transmission electron microscopy (TEM) images of sEV-containing pellets. Arrowheads indicate round vesicles. Scale bar, 200 nm. (**E**–**I**) Cortical neurons were treated with sEVs (5 μg/mL) purified from the indicated fibroblast cell lines, 4 h after plating. Neurons were fixed at 24 and 33 h, and neuronal morphology was examined after staining for Tuj1. (**E**) Representative images are shown. Arrowheads mark the longest neurite. Scale bar, 40 μm. (**F**–**I**) The length of the longest neurite (prospective axon; **F**), individual neurite/dendrite lengths (**G**), total dendrite length (longest neurite excluded; **H**), and total number of neurites (**I**) were quantified from a minimum of 90 neurons per condition from three independent experiments. Neurite lengths are plotted as a violin plot with values from each experiment distinctly colored and the median marked by a black line (**F**,**G**,**H**). The number of neurites is plotted as the average of the median ± SEM (**I**), where each dot represents the median from 30 neurons from one of the three independent experiments. Statistical significance: * *p* < 0.05, ** *p* < 0.01, *** *p* < 0.001 using one-way ANOVA with Dunnett’s post-test.

Dissociated primary mouse embryonic cortical neurons, when freshly plated in vitro (Figure S1A), give rise to short neurites, one of which grows rapidly to form an axon by 24–48 h, resulting in a polarized neuron [2,58]. Recent studies have demonstrated that exosomes isolated from mesenchymal stem cells can promote neurite outgrowth [18,19]. Thus, we sought to explore the role of fibroblast-derived EVs in neurite outgrowth. Following the guidelines of the International Society for Extracellular Vesicles (ISEV) [59], the isolated vesicles are hereafter referred to as sEVs. We first isolated and characterized sEVs from diverse mouse and human fibroblast cell culture supernatants, including mouse L cells, mouse embryonic fibroblasts (MEFs), human dermal fibroblasts (HDFn), normal human lung fibroblasts (NHLF), and BJ foreskin fibroblasts using differential centrifugation (Figure 1A). Analysis of the final pellet by immunoblotting revealed the presence of EV markers CD81, TSG101, and Flotillin1, and the absence of the endoplasmic reticulum (ER)-resident protein, calnexin (CNX) (Figure 1B). Particle size distribution analysis of the final pellet using nanoparticle tracking analysis (NTA) showed that the majority of particles were within a size range of 30–150 nm (Figure 1C), characteristic of sEVs [8]. Ultrastructural analysis by transmission electron microscopy (TEM) further showed intact round vesicles ranging in size from 30–150 nm in diameter, thus confirming the identity and structural integrity of the isolated sEVs (Figure 1D).

We next investigated the effect of the fibroblast-derived sEVs on neuronal morphology using dissociated E15.5-16.5 primary mouse embryonic cortical neurons. Neurons were treated 4 h after plating with sEVs for either 20 or 29 h and were fixed and stained with a Tuj1 antibody, which detects the neuron-specific β-III tubulin. Quantification of neurite lengths revealed that sEVs from all five fibroblast lines enhanced the length of the longest neurite (i.e., the prospective axon) from a median length of ~30 μm (in controls) to ~60 μm at 24 h and from ~40 μm (in controls) to ~75 μm at 33 h (Figure 1E,F). In contrast, the individual lengths (~10 μm) and the total combined length (~20 μm) of the remaining neurites, excluding the longest, referred to as minor neurites and corresponding to prospective dendrites, remained either unchanged or slightly reduced (Figure 1G,H). The total number of neurites was also unaltered by sEV treatment (Figure 1I).

Similarly, when neurons were treated with varying concentrations of sEVs (0.05–10 μg/mL) obtained from all five fibroblast cell lines, a dose-dependent increase in the length of the longest neurite at both 24 and 33 h was observed, with the maximal effect occurring at 5 μg/mL (Figure S1B–K). To verify that sEVs preferentially act on axons, neurons treated with sEVs from L cells were co-stained with the axonal marker Tau-1 and the dendritic marker MAP2. Quantification of Tau-1-positive axons confirmed that sEVs increased the length of axons (Figure S2A,B), yielding results similar to those obtained when the length of the longest neurite, independent of Tau-1 staining, was quantified in parallel (Figure S2C). Similar to the data observed in Figure 1G, the length of individual neurites (~10 μm) positive for the dendritic marker MAP2 was either unaffected or slightly reduced by sEVs (Figure S2D). Moreover, sEV treatment concordantly resulted in a significant increase in the percent of neurons with Tau-positive axons, a characteristic of a polarized neuron that was clearly evident within 24 h of plating (Figure S2E). Importantly, the increase in the percent of Tau-positive axons could be observed as early as 12 h after plating, indicating the rapid effect of sEVs (Figure S2F,G). Taken together, these results demonstrate that sEVs derived from a variety of fibroblast lines promote axon outgrowth and accordingly enhance the acquisition of a polarized phenotype.

The sEV-induced increase in axon outgrowth was lost when sEVs were incubated with detergent, which causes lysis of sEVs [60,61], or upon treatment with Proteinase K, which digests surface-accessible proteins (Figure S3A,B). This indicates that while the axon-promoting activity is not found inside the sEVs, intact sEVs are required for function, suggesting that the active proteins might be expressed on the surface of sEVs. Next, to rule out a potential role for contaminating cell membranes or protein aggregates in mediating the effects on neuronal morphology, we undertook two different and more stringent sEV isolation protocols, where the final ultracentrifugation (100,000× *g*) pellet of sEVs obtained from L cell supernatants was further subjected to either density gradient purification (Figure S3) or size exclusion chromatography (Figure S4). For density gradient separation, the resuspended pellet was applied to a discontinuous iodixanol gradient and then subjected to an additional ultracentrifugation (Figure S3C). Characterization of the floating fractions by immunoblotting demonstrated that Fractions 6–8 were positive for the EV markers, CD81 and Flotillin1 (Figure S3D), with the intensity of both markers peaking in Fraction 7 (Figure S3E). Of note, Fraction 9 had the highest amount of total protein but very low levels of EV markers (Figure S3E), indicating a successful enrichment of EV particles in Fractions 6–8. Particle size distribution analysis showed that the number of particles peaked at approximately 100 nm, which is characteristic of sEVs, and that Fraction 7 had the highest number of particles (Figure S3F). Moreover, Fraction 7 (F7) had the highest particle/protein ratio (Figure S3G), a parameter that is widely used to measure the purity of EVs [62,63]. Treatment of dissociated neurons with these purified sEVs from the F7 fraction revealed that the length of the longest neurite (i.e., the prospective axon) was increased to levels similar to that observed for the initial ultracentrifugation pellet (sEVs) at all time points (Figure S3H,I).

Next, we used size exclusion chromatography as an alternative approach to purify sEVs from the ultracentrifugation (100,000× *g*) pellets (Figure S4A). Characterization of the isolated fractions revealed the presence of the EV markers, CD81, Flotillin1, and TSG101, but not the ER protein, calnexin (CNX) in fractions 7–12 (Figure S4B), with particle size peaking at a diameter of approximately 100 nm with fraction F7 having the highest particle/protein ratio (Figure S4C,D). Treatment of dissociated neurons with F7 purified sEVs revealed that the length of the longest neurite (i.e., the prospective axon) was increased to levels similar to that observed for the initial ultracentrifugation pellet (sEVs) at both 24 and 33 h (Figure S4E,F), as observed previously (Figure 1E,F and Figure S1). Of note, outgrowth induced by sEVs isolated using either purification method was easily detected as early as 12 h (i.e., 8 h after sEV addition), highlighting the rapid nature of the response (Figures S3H,I and S4E,F).

Altogether, these observations suggest that the sEVs isolated by ultracentrifugation (100,000× *g* pellet) are responsible for promoting axon outgrowth. Since the responses induced by these pelleted sEVs were similar to those achieved after more extensive purifications, we used this ultracentrifuged pellet as a source of sEVs for all subsequent experiments.

### 3.2. The PCP Pathway Is Required for sEV-Induced Growth of the Prospective Axon

**Figure 2:**
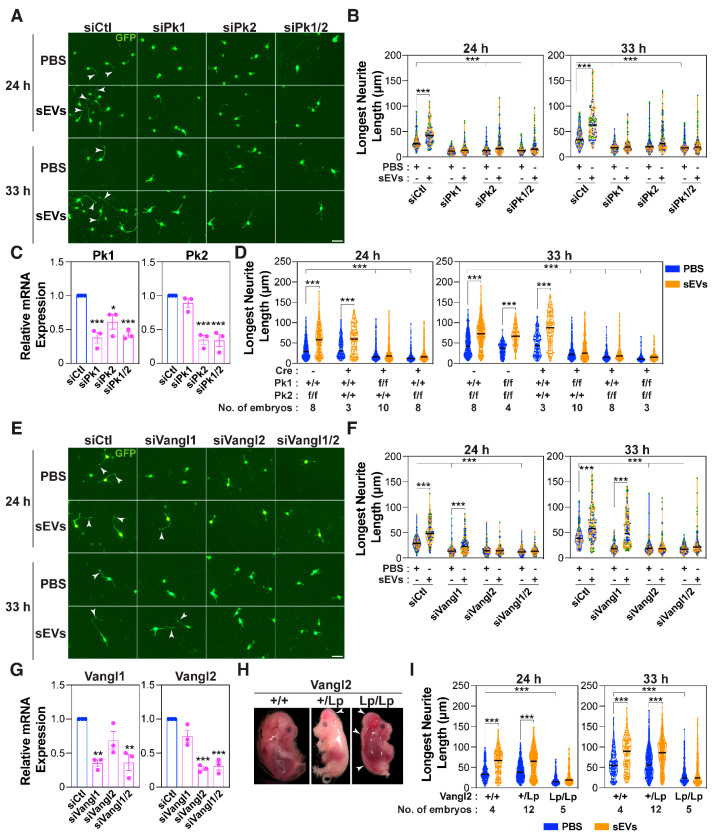
sEVs promote the growth of the prospective axon. The PCP components, Pk1/2 and Vangl2, are required for sEV-induced growth of the longest neurite. (**A**–**C**,**E**–**G**) Pk1/2 and Vangl2 promote sEV-induced neurite outgrowth. Dissociated E15.5-16.5 mouse cortical neurons were electroporated with siRNA against Pk1 (siPk1) and Pk2 (siPk2) (**A**–**C**), or Vangl1 (siVangl1) and Vangl2 (siVangl2) (**E**–**G**) individually or in combination or with siControl (siCtl) along with a GFP-expressing plasmid and were treated with sEVs (5 μg/mL) from L cells, 4 h after plating. Neurons were fixed at 24 and 33 h, and neuronal morphology was examined in GFP-positive neurons. (**A**,**E**) Representative images are shown. Arrowheads mark the longest neurite. Scale bar, 40 μm. (**B**,**F**) The length of the longest neurites was quantified. (**C**,**G**) Knockdown efficiency for Pk1/Pk2 (**C**) and Vangl1/2 (**G**) was determined in GFP-positive neurons isolated by FACS. Relative mRNA expression was determined by qPCR. (**D**,**I**) Pk1/2 and Vangl2 promote sEV-induced neurite outgrowth in mutant mouse models. Cortical neurons (E15.5-16.5) were isolated from Pk1 and Pk2 conditional knockout mice obtained by crossing *Pk* floxed mice with a *Nestin-Cre* line (**D**) or *Vangl2* mutant littermates obtained by crossing heterozygous loop-tail mutants (*Vangl2^+/Lp^*) (**I**). Neurons were treated with sEVs from L cells, 4 h after plating, fixed at 24 and 33 h, and morphology examined in Tuj1 stained neurons. The length of the longest neurite was quantified from 40 neurons per embryo, and the total number of embryos analyzed is indicated below the genotypes. Violin plots with the median marked by a black line are shown. (**H**) Loop-tail embryos (E15.5-E16.5) exhibit an open neural tube. Representative images are shown from a minimum of four independent experiments. Arrowheads mark the open neural tube. In siRNA experiments, neurite lengths are quantified from a minimum of 90 neurons per condition from three independent experiments and plotted as a violin plot with values from each experiment distinctly colored and the median marked by a black line (**B**,**F**). For qPCR plots, data is presented as the mean ± SEM from 3 independent experiments (**C**,**G**). Statistical significance: * *p* < 0.05, ** *p* < 0.01, *** *p* < 0.001 using one-way ANOVA with Dunnett’s post-test (**C**,**G**), or two-way ANOVA with Tukey’s post-test (**B**,**D**,**F**,**I**).

Previous work has demonstrated that in cancer cells, motility induced by exogenously added fibroblast-derived exosomes is mediated by the Wnt planar cell polarity pathway (PCP) in recipient cells [25]. Prickle (Pk) and Vangl, core PCP components, distribute asymmetrically to the proximal side in planar polarized epithelial cells [29,33] and are also known to modulate neuronal morphology [64,65]. This prompted us to investigate whether core PCP components might be required for sEV-induced axon outgrowth. To study this, we used a combination of siRNA-mediated knockdown and mouse genetic models to explore the role of these core PCP components in dissociated primary cortical neurons. There are two Prickle genes, *Prickle 1* and *Prickle 2* (*Pk1* and *Pk2*), expressed in cortical neurons (Figure S5A). To test for the requirement for Prickle, neurons were co-transfected with siRNAs targeting *Pk1* and/or *Pk2* along with a plasmid encoding GFP to visualize transfected cells. Analysis of GFP-positive neurons in which expression of Pk1 and Pk2 alone or together was abrogated revealed that neurons were comprised solely of minor neurites (mean length ~10 μM), none of which were elongated upon treatment with sEVs (Figure 2A,B). Knockdown efficiency was confirmed by qPCR after FACS sorting for GFP-positive neurons (Figure 2C). As an alternative to this transient approach, we explored the consequences on neuronal morphology using dissociated neurons isolated from mice with genetic loss of *Pk1* or *Pk2*. While *Pk2* knockout mice are viable [66], *Pk1* knockout mice display early embryonic lethality [67]. Thus, we generated mice with single floxed *Pk* alleles (*Pk1^f/f^* and *Pk2^f/f^*) using targeted homologous recombination (Figure S5B), which were then crossed with each other to obtain mice with double (*Pk1^f/f^;Pk2^f/f^*) floxed *Pk* alleles. Both single and double floxed mice, after removal of the neomycin cassette, were viable, fertile, and exhibited no overt anatomical defects. Conditional knockout mice were then generated by crossing with *Nestin-Cre* mice, which express Cre under the *Nestin* promoter, to achieve loss of *Pk1/2* in the developing brain with the caveat that Nestin expression is not restricted to neurons only. Loss of Pk1/2 expression in the cortex of *Pk1^f/f^;Pk2^f/f^;Nestin-Cre* E15.5 embryos was confirmed by immunofluorescence microscopy using Pk1 and Pk2 antibodies (Figure S5C,D). Consistent with the siRNA results, analysis of cortical neurons (E15.5-16.5) isolated from mice with a *Nestin-Cre* conditional deletion of either *Pk1* alone, *Pk2* alone, or both (*Pk1^f/f^;Pk2^+/+^;Nestin-Cre, Pk1^+/+^;Pk2^f/f^;Nestin-Cre*, or *Pk1^f/f^;Pk2^f/f^;Nestin-Cre*, respectively) revealed the presence of only short neurites that failed to elongate in response to sEV treatment, in contrast to the *Pk1^+/+^;Pk2^f/f^ or Pk1^+/+^;Pk2^+/+^;Nestin-Cre* controls (Figure 2D and Figure S5E).

We next explored the contribution of the core PCP tetraspanin proteins, Vangl1 and Vangl2, which regulate diverse aspects of neuronal development [65]. Abrogation of Vangl2 expression using siRNAs strongly decreased sEV-mediated induction of outgrowth of the longest neurite (Figure 2E–G). Interestingly, in the case of siVangl1, while the length of the longest neurite was reduced, sEVs maintained the ability to promote elongation (Figure 2E–G), suggesting that Vangl2, but not Vangl1, is important for the sEV-mediated effects. Loop-tail mice harbor an S464N point mutation in the *Vangl2* allele [68,69] that results in reduced Vangl2 protein levels and defective translocation to the plasma membrane [70,71,72]. Morphological examination showed that the heterozygous loop-tail embryos (*Vangl2^+/Lp^*) exhibited a curly tail with partial or no neural tube defects, whereas homozygous loop-tail embryos (*Vangl2^Lp/Lp^*) exhibited a completely open neural tube (Figure 2H) as previously reported [69]. Consistent with the siRNA results, dissociated cortical neurons isolated from E15.5-16.5 *Vangl2^Lp/Lp^* embryos, but not wild-type or heterozygotes (Lp/+) displayed short neurites (~10 μM in length) and a loss of sEV-induced outgrowth of the longest neurite at both 24 and 33 h (Figure 2I and Figure S5F).

Smurf1 and Smurf2 are E3 ubiquitin ligases that modulate PCP signaling to control convergent extension, cell protrusion, and motility [25,49,73,74] and have also been shown to play a key role in regulating neuronal morphology [75,76,77]. Since cortical neurons express both Smurf1 and Smurf2 as determined by RNA sequencing analysis (Figure S5A), we next explored whether Smurfs might have a role in sEV-induced axon outgrowth. For this, we examined the contribution of Smurf1 and/or Smurf2 using dissociated neurons isolated from Smurf knockout mice. *Smurf1^+/−^;Smurf2^+/−^* double heterozygotes [49] were crossbred to generate offspring with varying Smurf1/2 genotypes. Analysis of dissociated cortical neurons at a later timepoint revealed that complete loss of either Smurf1 alone (*Smurf1^−/−^;Smurf2^+/+^*) or Smurf2 alone (*Smurf1^+/+^;Smurf2^−/−^*) yielded neurons that harbored only short neurites (~10 μM in length) and that sEV-induced outgrowth of the longest neurite was prevented, in contrast to wild-type or heterozygote (*Smurf1^+/−^;Smurf2^+/−^*) littermates (Figure S5G,H). These results suggest that Smurf1 and Smurf2 act in a non-redundant manner or that a minimal level of combined Smurf1/2 activity is required for neurite outgrowth.

**Figure 3:**
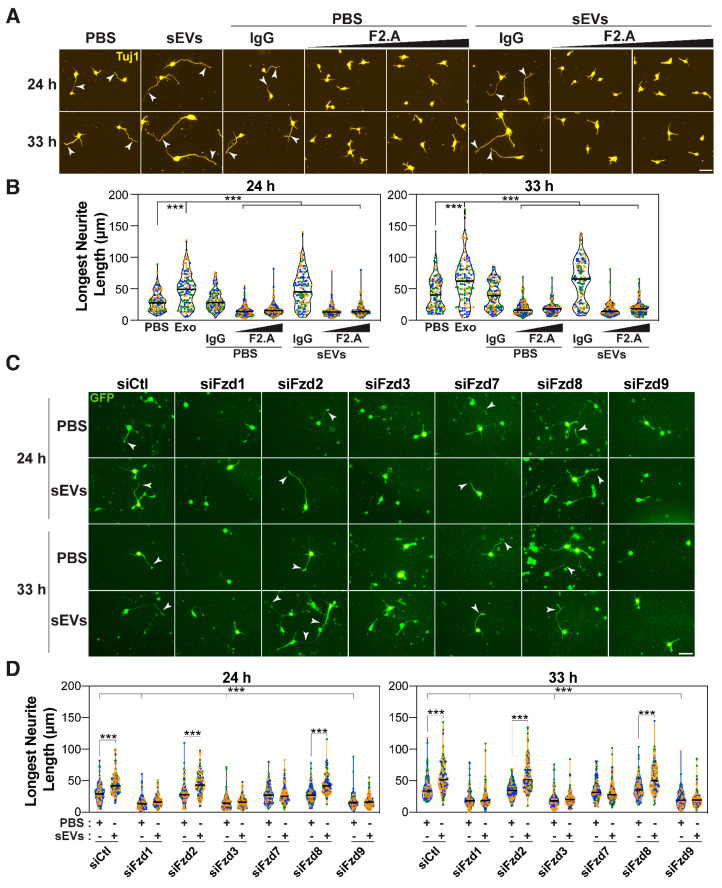
sEVs promote the growth of the prospective axon. (**A**–**D**) Dissociated E15.5-16.5 mouse cortical neurons were treated with sEVs (5 μg/mL) from L cells, 4 h after plating. (**A**,**B**) Neurons were co-treated with IgG or an Fzd blocking antibody, F2.A (at 50 nM and 100 nM), along with sEVs. (**C**,**D**) Neurons were electroporated with siRNA against Fzds (siFzds) or siControl in combination with a GFP-expressing plasmid prior to the addition of sEVs. Neurons were fixed at 24 and 33 h, and neuronal morphology was examined in Tuj1 stained (**A**,**B**) or GFP-positive neurons (**C**,**D**), with representative images shown. Arrowheads mark the longest neurite. Scale bar, 40 μm. (**B**–**D**) The length of the longest neurite was quantified. Neurite lengths are quantified from a minimum of 90 neurons per condition from three independent experiments and plotted as a violin plot with values from each experiment distinctly colored, and the median marked by a black line (**B**,**D**). Statistical significance: *** *p* < 0.001 using one-way ANOVA with Dunnett’s post-test (**B**), or two-way ANOVA with Tukey’s post-test (**D**).

**Figure 4:**
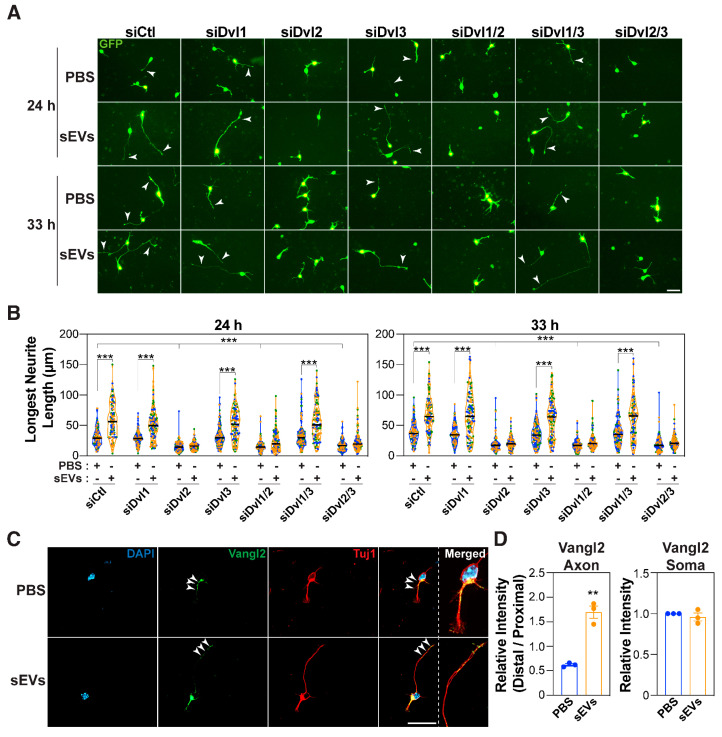
sEVs promote the growth of the prospective axon. (**A**,**B**) Dissociated E15.5-16.5 mouse cortical neurons were treated with sEVs (5 μg/mL) from L cells, 4 h after plating. Neurons were electroporated with siRNA against Dvls (siDvl) or siControl in combination with a GFP-expressing plasmid prior to the addition of sEVs. Neurons were fixed at 24 and 33 h, and neuronal morphology was examined in GFP-positive neurons, with representative images shown (**A**). Arrowheads mark the longest neurite. Scale bar, 40 μm. (**B**) The length of the longest neurite was quantified. (**C**,**D**) sEVs promote localization of Vangl2 to the distal axon. Cortical neurons were treated with sEVs from L cells, 4 h after plating and fixed after 24 h. Representative confocal images of neurons stained with DAPI (blue), Vangl2 (green), and Tuj1 (red) are shown. Arrowheads mark the Vangl2 localization. Scale bar, 50 μm. (**D**) The ratio of distal/proximal Vangl2 intensity and the relative intensity of Vangl2 in the soma is quantified from 30 neurons from three independent experiments. Neurite lengths are quantified from a minimum of 90 neurons per condition from three independent experiments and plotted as a violin plot with values from each experiment distinctly colored, and the median marked by a black line (**B**). For Vangl2 plots, data are presented as the mean ± SEM from 3 independent experiments (**D**). Statistical significance: ** *p* < 0.01, *** *p* < 0.001 using unpaired *t*-test (**D**), or two-way ANOVA with Tukey’s post-test (**B**).

Both PCP and canonical β-catenin signaling are initiated upon binding of Wnt ligands to one of 10 members of the seven-pass transmembrane Frizzled family of receptors (Fzd) and are then propagated intracellularly through one of three Dishevelleds (Dvls) [29,47,78,79,80,81]. Thus, we next explored which Fzds and Dvls are required for sEV-mediated axon outgrowth. Cortical neurons treated with a pan-Fzd blocking antibody (F2.A) that recognizes multiple Fzds, including Fzd1, 2, 4, 5, 7, and 8 [51,82], displayed only minor neurites that failed to elongate in response to sEVs (Figure 3A,B). Treatment of cortical neurons with F2.A at a later time point had no effect on the axon length; however, the sEV-induced increase in axon length was impaired, indicating that Fzds are required to mediate sEV effects (Figure S6A,B). Mouse E15.5-16.5 cortical neurons express Fzd1, 2, 3, 7, 8, and 9, with Fzd1 and Fzd3 displaying the highest levels (Figure S5A). Thus, we next explored the individual requirement for these receptors using siRNAs. Quantification revealed that the individual loss of Fzd1, 3, or 9 yielded neurons with minor neurites that failed to elongate in response to sEVs, while the loss of Fzd7 only blocked sEV-mediated outgrowth (Figure 3C,D and Figure S6C). Loss of Fzd2 or Fzd8 had no effect, though equivalent knockdown efficiency was achieved (Figure S6C). These results indicate that multiple but specific Fzds (Fzd1, 3, and 9) are required for basal elongation, whereas only Fzd7 is specifically required for sEV-mediated outgrowth of the prospective axon. All three Dishevelleds (Dvl1, 2, and 3) are expressed in cortical neurons, with Dvl1 being expressed at the highest level (Figure S5A). Analysis of the requirement for Dvls using siRNAs revealed that only Dvl2 was required for sEV-induced outgrowth (Figure 4A,B and Figure S7A).

Individual Fzds and Dvls function in both PCP and β-catenin pathways in a complex and highly redundant manner, thus it is challenging to associate specific Fzds/Dvls with a particular pathway [79,80,83]. However, it is worth noting that evidence of Fzd7 and Dvl2, both of which we observed to be specifically required for sEV-induced growth of the prospective axon, has been reported to function in PCP pathways [38,84]. Taken together, these observations suggest that core PCP components are required for basal neurite elongation and that they are required for sEV-induced growth of the longest neurite.

### 3.3. Vangl2 Localizes Asymmetrically During sEV-Induced Growth of the Longest Neurite

Prior work has revealed that Wnt5a-mediated activation of the PCP pathway in dissociated commissural neurons results in relocalization of Vangl2 to the growth cone [85]. Thus, we next explored whether sEVs might similarly promote relocalization of Vangl2.

In control (PBS) or sEV-treated embryonic cortical neurons, Vangl2 was distributed in both the somal membrane and in the axon, but in distinct patterns (Figure 4C). Quantification of Vangl2 distribution within the axon shaft revealed that in controls, a preferential localization in the proximal half of the axon was observed (Figure 4D), which was independent of axon length (Figure S7B). Remarkably, in neurons treated with sEVs for 20 h, Vangl2 relocalized to the distal end of the axon (distal to proximal intensity ratio of ~1.6 versus ~0.6 in controls) (Figure 4D). No overt change in Vangl2 localization in the somal membrane was observed. This relocalization of Vangl2 to the distal axon suggests that sEVs engage PCP components to promote axon growth.

### 3.4. Neuronal Wnts Mediate sEV-Induced Growth of the Longest Neurite

**Figure 5:**
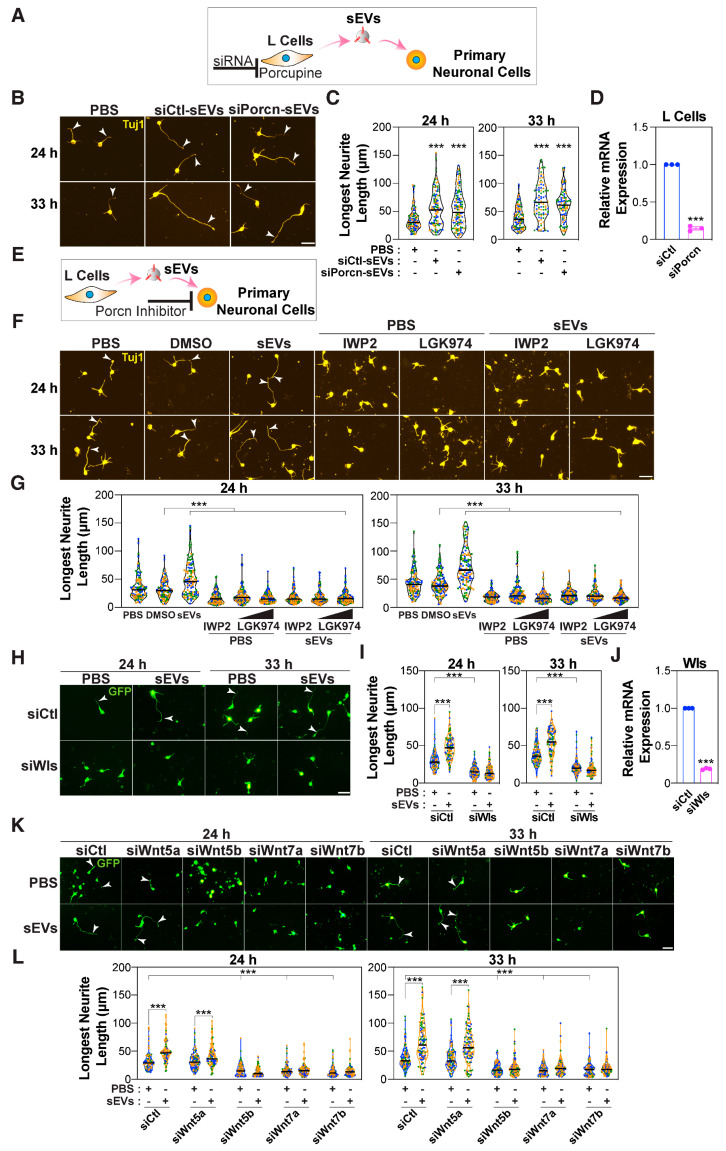
Neuronal Wnts mediate sEV-induced growth of the longest neurite. (**A**,**E**) Schematics illustrating the experimental setup. (**A**–**G**) Dissociated E15.5-16.5 mouse cortical neurons were treated with sEVs isolated from L cells transfected with siCtl or siPorcupine (**A**–**D**) or with sEVs isolated from regular L cells (**E**–**G**). Neurons were treated with PBS as a control and with Porcupine (Porcn) inhibitors, IWP2 (10 μM) or LGK974 (1 and 5 μM) (**E**–**G**), 4 h after plating and co-incubated with sEVs. (**H**–**L**) Cortical neurons were electroporated with siRNAs against Wls (siWls) (**H**,**I**) or Wnts (siWnts) (**K**,**L**) or siControl (siCtl) along with a GFP-expressing plasmid and then treated with sEVs (5 μg/mL) from L cells, 4 h after plating. Neurons were fixed at 24 and 33 h, and neuronal morphology was examined in Tuj1 stained neurons (**B**,**C**,**F**,**G**) or GFP-positive neurons for siRNA experiments (**H**,**I**,**K**,**L**). Representative images (**B**,**F**,**H**,**K**) and quantifications of the longest neurite (**C**,**G**,**I**,**L**) are shown. Arrowheads mark the longest neurite. Scale bar, 40 μm. (**D**,**J**) Knockdown efficiency for Porcupine (**D**) and Wls (**J**) was determined in L cells and GFP-positive neurons isolated by FACS, respectively. Relative mRNA expression was determined by qPCR. Neurite lengths are quantified from a minimum of 90 neurons per condition from three independent experiments and plotted as a violin plot with values from each experiment distinctly colored and the median marked by a black line (**C**,**G**,**I**,**L**). For all other plots, data is presented as the mean ± SEM from three independent experiments (**D**,**J**). Statistical significance: *** *p* < 0.001 using unpaired *t*-test (**D**,**J**), one-way ANOVA with Dunnett’s post-test (**C**,**G**), or two-way ANOVA with Tukey’s post-test (**I**,**L**).

Wnts, including Wnt5a, Wnt7a, and Wnt7b, can promote axon elongation, neurogenesis, and axon guidance, respectively, via the PCP pathway [44,45]. As exosomes are known to carry Wnts [40,86,87], we next explored whether the presence of Wnt ligands in isolated sEVs could contribute to the observed outgrowth of neurites. We first abrogated the expression of Porcupine, an acyltransferase essential for Wnt lipidation and secretion [88,89], in the exosome-producing L cells using siRNAs and isolated the sEVs (Figure 5A). Abrogation of Porcupine expression had no effect on the overall number of sEVs secreted or on the levels of the EV markers, CD81, Flotillin1, and TSG101, as indicated by immunoblotting (Figure S7C–F). Moreover, ultrastructural analysis by TEM revealed intact round, exosome-like vesicles in both the control and Porcupine knockdown samples (Figure S7G). When applied to cortical neurons, sEVs isolated from Porcupine-deficient L cells were as active as control sEVs in promoting outgrowth of the longest neurite (Figure 5B–D). Immunodetection of endogenous Wnt proteins in wild-type L cells is challenging; thus, we confirmed that siPorcupine inhibits Wnt secretion using L cells stably expressing Wnt5a (L-Wnt5a) (Figure S7H–J). These results indicate that the activity of L cell-derived sEVs was not due to the presence of Wnts within the sEVs.

We next sought to determine whether L cell-secreted exosomes might modulate autocrine Wnt signaling, a process in which secreted Wnts bind to cell-surface receptors and activate Wnt signaling in neurons to promote neurite outgrowth. To investigate this, we blocked the ability of neurons to secrete Wnts, thus attenuating the autocrine signaling, by chemically inhibiting the activity of Porcupine using IWP2 or LGK974 [90,91] (Figure 5E). As endogenous Wnt proteins are difficult to detect in neurons, we confirmed that IWP2 blocked Wnt secretion using L-Wnt5a cells (Figure S7K), as described above for siPorcupine (Figure S7H–J). We observed that treatment of cortical neurons with Porcupine inhibitors 4 h after plating prevented sEV-mediated neurite outgrowth (Figure 5F,G). In contrast, treatment with IWP2 at 24 h after plating had no effect on basal axon lengths but impaired the sEV-induced axon outgrowth, thus indicating that neuronal Wnts are required to mediate the effect of sEVs (Figure S8A,B). The Wnt carrier protein, Wntless (Wls), is required for Wnt secretion [92], and abrogating expression of Wls using siRNAs similarly prevented sEV-induced neurite outgrowth (Figure 5H–J). Thus, blocking pathways essential for Wnt secretion prevents sEV-induced growth of the longest neurite, indicating that neuronally expressed endogenous Wnts are necessary for sEV function.

Analysis of RNA sequencing results revealed that cortical neurons express Wnt5a, Wnt5b, Wnt7a, and Wnt7b (Figure S5A). We verified by qPCR that these Wnts were expressed in neurons and that expression levels remained constant throughout the time frame of the experiment (Figure S8C). Thus, to determine which Wnts are important for neurite outgrowth, the expression of each Wnt was individually abrogated using siRNAs (Figure S8D). Neurons lacking Wnt5b, Wnt7a, or Wnt7b, but not Wnt5a, were comprised solely of minor neurites, none of which elongated upon treatment with sEVs (Figure 5K,L). Altogether, these observations suggest that exogenously added sEVs might engage endogenous Wnts (Wnt5b, Wnt7a, and Wnt7b) to promote growth of the longest neurite.

### 3.5. sEVs Associate with Wnts During Neurite Outgrowth

**Figure 6:**
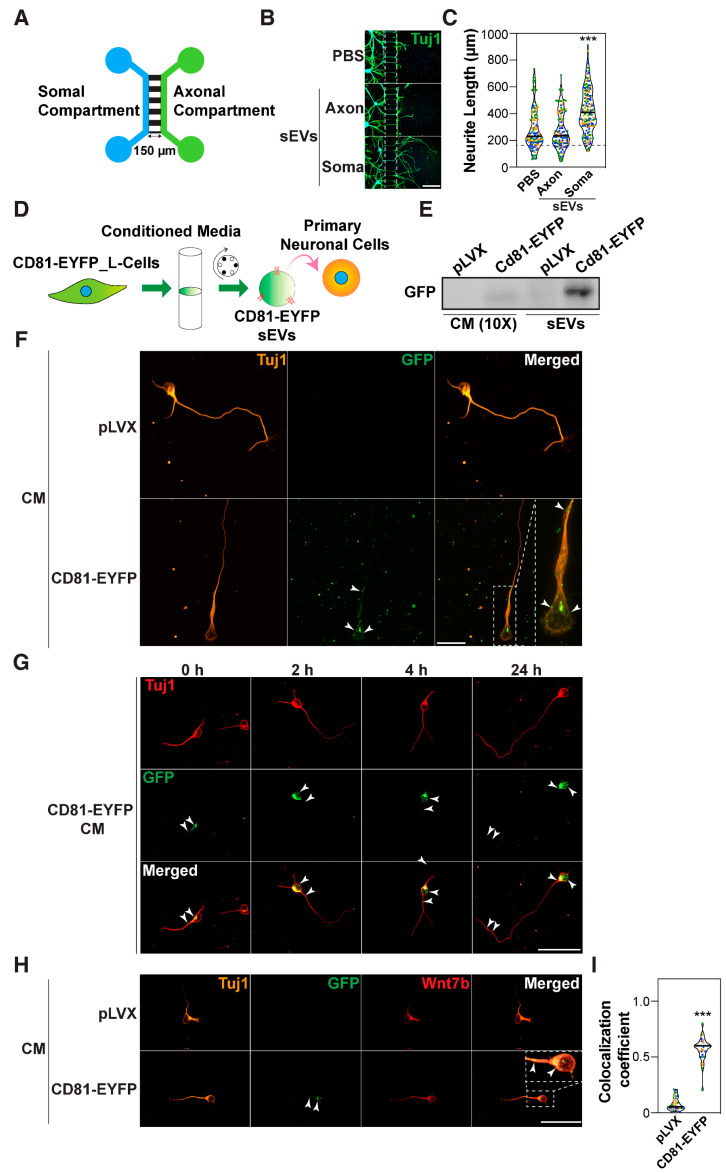
sEVs promote neurite elongation and can colocalize with Wnt7b. (**A**–**C**) sEVs promote neurite elongation. (**A**) A schematic illustration of the two-compartment Xona microfluidic device. The somal compartment is connected to the axonal compartment through a 150 μm microgroove. (**B**) Cortical neurons (E15.5-16.5) were seeded in the somal compartment and cultured for 5 days prior to the addition of L cell-derived sEVs in either the somal or axonal compartment. Neurons were fixed 24 h later, and neuronal morphology was examined in Tuj1 stained neurons. Representative images are shown. Scale bar, 200 μm. (**C**) The length of the neurites growing in the microgroove and emerging in the axonal compartment was quantified for a minimum of 90 neurites. A dotted line marks both ends of the microgroove (150 μm). (**D**–**I**) sEVs can be internalized by neurons and colocalize with Wnts. Cortical neurons were treated with 10X concentrated conditioned media (CM) from L cells stably expressing CD81-EYFP, 4 h after plating for 29 h (**F**) or 24 h after plating for 30 min (**G**–**I**). In panel (**G**), after 30 min of treatment, neurons were washed and subsequently treated with regular complete media for 0, 2, 4, and 24 h. Representative images of neurons immunostained with GFP and Tuj1 (**F**,**G**) or GFP, Tuj1, and Wnt7b (**H**) are shown. Dashed boxes (**H**) indicate higher magnification of neurons. Arrowheads mark GFP puncta of internalized sEVs. Scale bar, 20 μm (**F**) or 40 μm (**G**,**H**). (**E**) Characterization of sEVs. The concentrated CM (10X) and sEV pellet from L cells were immunoblotted with anti-GFP antibody. (**I**) Pearson’s colocalization coefficient for (**H**). Neurons were identified using Tuj1 as a reference channel, and the colocalization coefficient was quantified using Nikon NIS-Elements software. Images (**F**,**G**,**H**) and the quantification (**I**) are representative of 30 neurons from 3 independent experiments. In all the violin plots, values are distinctly colored for each experiment, and the median is marked by a black line. Statistical significance: *** *p* < 0.001 using unpaired *t*-test (**I**) or one-way ANOVA with Dunnett’s post-test (**C**).

To explore sEV uptake in neurons, we used a compartmentalized microfluidic chamber device [93] comprised of a set of proximal compartments, where neurons are seeded, and a set of distal compartments where axons emerge. The compartments are connected by a microgroove barrier that enables a gradient-based fluidic separation due to the hydrostatic pressure (Figure 6A). Plated neurons were grown for 5 days to allow axons to reach the distal compartment. sEVs were added to either compartment for 24 h, and then neurons were fixed and stained for Tuj1. Addition of sEVs to the proximal compartment containing the cell bodies but not to the distal compartment containing axon tips promoted elongation of neurites (Figure 6B,C).

To directly visualize sEV uptake, we used conditioned media (CM) from L cells stably expressing CD81-EYFP as a source of EYFP-labelled sEVs [25]. Immunoblotting of both the conditioned media (CM) and the sEV pellet revealed the presence of EYFP using an anti-GFP antibody (Figure 6D,E), indicating successful integration of the CD81-EYFP fusion protein in sEVs. Neurons were treated with the CM from either control or CD81-EYFP-expressing L cells for 29 h. Immunostaining followed by confocal microscopy revealed that the majority of GFP-positive neurons displayed abundant GFP puncta within the soma as well as occasional staining in the axon, suggesting that sEVs were internalized (Figure 6F). Next, neurons were incubated with CM containing CD81-EYFP-labelled sEVs for 30 min, washed, and subsequently chased in regular CM media for varying times prior to fixation. We found that sEVs were internalized by neurons within the 30 min exposure time, and that the internalized sEVs were found both in the soma and in axons throughout the time course (0–24 h) (Figure 6G). Thus, sEVs are rapidly (within 30 min) taken up by neurons and can be detected as long as 24 h later.

In cancer cells, internalized exosomes acquire endogenous Wnts to activate PCP signaling [25]. Given that sEVs were internalized in neurons, we next investigated whether the internalized sEVs could associate with the Wnts. Cortical neurons were electroporated with HA-tagged Wnt7b (Wnt7b-HA) or an empty vector control, and 24 h after plating, were treated with EYFP-labelled sEVs for 30 min. Immunostaining with anti-HA and GFP antibodies showed colocalization of Wnt7b-HA and sEVs in the soma (Figure S8E). Next, we examined whether sEVs were colocalized with endogenous Wnt7b, the ligand most highly expressed in neurons. Neurons were treated with 10X concentrated CM obtained from either control or CD81-EYFP expressing L cells for 30 min and then fixed and immunostained with anti-GFP and anti-Wnt7b antibodies, whose specificity was first confirmed by immunofluorescence microscopy (Figure S8F). This analysis revealed colocalization of Wnt7b and sEVs in both the soma and axon (Figure 6H,I). Given that internalized sEVs tend to colocalize with Wnt7b, and that endogenous Wnts are required for sEV-induced outgrowth, our findings suggest that internalized sEVs may acquire neuronally expressed Wnt7b to promote axon outgrowth.

### 3.6. sEVs from Neurons and Astrocytes Do Not Promote Neurite Outgrowth

**Figure 7:**
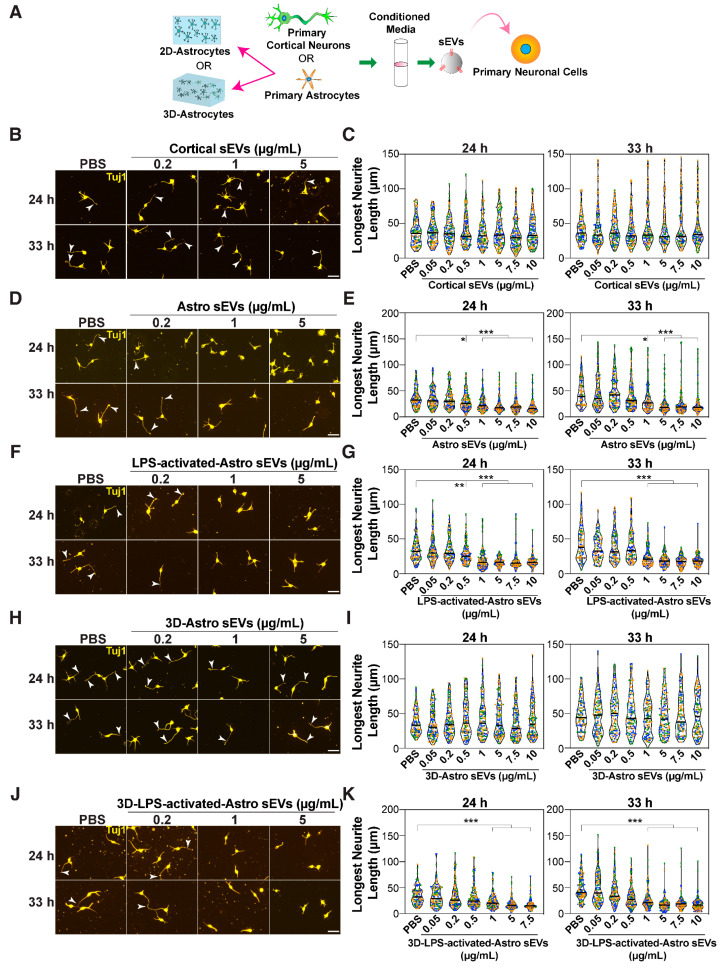
sEVs from neurons and astrocytes do not promote the growth of the longest neurite. (**A**) A schematic illustration of the experimental set up. Dissociated E15.5-16.5 cortical neurons were treated with sEVs purified from primary cortical neurons or primary astrocytes. (**B**–**K**) Cortical neurons were treated with various concentrations (0.05–10 μg/mL) of sEVs purified from cortical neurons (**B**,**C**), astrocytes (**D**,**E**), LPS-activated astrocytes (**F**,**G**), 3D-astrocytes grown in collagen gel (**H**,**I**), and LPS-activated 3D-astrocytes (**J**,**K**) 4 h after plating. Neurons were fixed at 24 and 33 h, and neuronal morphology was examined in Tuj1 stained neurons. Representative images (**B**,**D**,**F**,**H**,**J**) and quantifications (**C**,**E**,**G**,**I**,**K**) are shown. Arrowheads mark the longest neurite. Scale bar, 40 μm. Neurite lengths are quantified from a minimum of 90 neurons per condition from 3 independent experiments and plotted as a violin plot, with values from each experiment distinctly colored and the median marked by a black line. Statistical significance: * *p* < 0.05, ** *p* < 0.01, *** *p* < 0.001 using one-way ANOVA with Dunnett’s post-test.

In the brain, exosomes mediate glia-neuron communication during developmental processes and pathogenesis [14,15,16,17]. Neurons and astrocytes are the two major cell types in the CNS and are known to secrete exosomes [14]. Thus, we sought to determine whether exosomes released from neurons or astrocytes also modulate neurite outgrowth. To examine this, we first isolated and tested sEVs produced by primary embryonic cortical neurons (Figure 7A). sEV pellets were positive for EV markers and displayed the typical 30–150 nm diameter size range (Figure S9A,B). However, in contrast to fibroblast-derived sEVs, these sEVs did not promote neurite outgrowth when added to neurons at any dose tested, including at concentrations much higher (0.05–10 μg/mL) than those required for fibroblast-derived sEV effects (Figure 7B,C). Thus, sEVs exogenously secreted from neurons do not promote neurite outgrowth.

Astrocytes support many physiological functions of neurons, including synapse development and neuronal activity, whereas reactive astrocytes produced during injury or aging [94] contribute to the production of the inhibitory environment that prevents axon regeneration [95,96,97,98]. Thus, we next investigated the role of astrocyte-derived exosomes in neurite outgrowth (Figure 7A). For this, we isolated primary mouse astrocytes from newborn pups at postnatal day (P) 1–4 and confirmed their identity by positive staining for GFAP (Figure S9C) and negative staining for the neuronal marker Tuj1 (Figure S9C).

Reactive astrocytes were generated by treating isolated astrocytes with bacterial lipopolysaccharide (LPS), which induced upregulation of the expression of proinflammatory cytokines, including TNF-α, iNOS-2, IL-1β, and IL-6, as determined by qPCR (Figure S9D), confirming astrocyte activation. sEVs were purified from control and LPS-activated astrocytes using differential centrifugation, and the presence of sEVs in pellets was confirmed by immunoblotting for CD81, TSG101, and Flotillin1, and by confirming particle diameters using NTA (Figure S9E,F). Interestingly, treatment of neurons with varying doses of sEVs isolated from either control or LPS-activated astrocytes inhibited neurite outgrowth (Figure 7D–G). As tissue culture plastic is much stiffer than the normal in vivo environment and can stimulate partial activation of astrocytes [99], we next cultured astrocytes in a soft 3D-collagen gel, hereafter referred to as 3D-astrocytes. GFAP expression is higher in activated astrocytes [100], and accordingly, astrocytes grown on a 2D-plastic surface showed much higher GFAP staining compared to those cultured in the 3D-collagen gel (Figure S9G). Moreover, treatment of 3D-astrocytes with LPS enhanced GFAP expression (Figure S9H). Next, sEVs were isolated from control and LPS-activated 3D-astrocytes and were confirmed to display typical exosome size using NTA (Figure S9I–K). Remarkably, sEVs isolated from 3D-astrocytes had no effect on neurite outgrowth (Figure 7H,I), whereas sEVs from LPS-activated 3D-astrocytes inhibited neurite outgrowth (Figure 7J,K). Thus, our results indicate that while reactive astrocytes inhibit neurite outgrowth, control, unstimulated astrocytes grown in a soft environment do not alter neuronal morphology.

**Figure 8:**
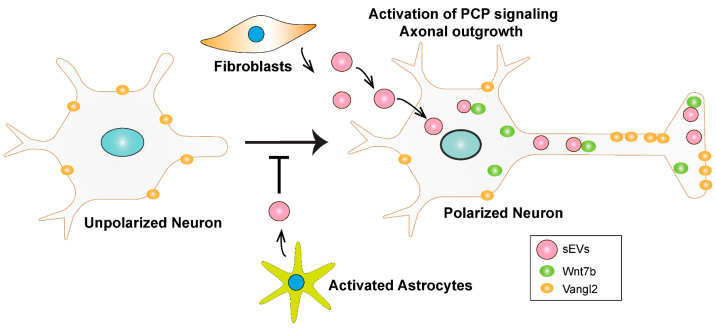
A model depicting the effect of sEVs in promoting axon outgrowth and polarized neuronal morphology through Wnt-PCP signaling. sEVs secreted by L cells engage Wnt-PCP signaling in neurons to promote axon outgrowth that results in the acquisition of a polarized neuronal morphology. sEVs induce a shift in Vangl2 localization towards the distal axon. sEVs can be internalized by neurons and can colocalize with Wnt7b to promote the growth of the prospective axon. In contrast to fibroblast-derived sEVs, those isolated from activated astrocytes inhibit neurite outgrowth.

Taken together, these findings suggest that fibroblast-derived sEVs can promote neurite outgrowth, and this ability is not a general property of sEVs. Collectively, the data support a model in which sEVs engage Wnt-PCP signaling in neurons to promote axon outgrowth and a polarized morphology. We suggest a novel mechanism whereby internalized sEVs, which tend to colocalize with Wnt ligands such as Wnt7b, induce relocalization of the core PCP component, Vangl2, from the soma to the distal part of the axon and thereby promote axon outgrowth (Figure 8).

## 4. Discussion

Exosomes are increasingly being studied for therapeutic applications [101,102]. Exosomes from diverse sources are shown to modulate neurite outgrowth, but how fibroblast-derived exosomes act to specifically affect neuronal morphology remains unclear. Here, we show that fibroblast-derived sEVs can engage Wnt-PCP signaling in neurons and thereby promote axon outgrowth and acquisition of a polarized neuronal morphology. We demonstrate that sEVs are internalized via the soma in neurons and can colocalize with endogenous Wnt7b and that sEVs induce relocalization of the core PCP component, Vangl2, to the distal region of the axon. Thus, our work demonstrates that sEVs can engage Wnt-PCP signaling in neurons to promote axon outgrowth.

Exosomes derived from diverse sources harbor the ability to promote axon or dendrite elongation. For instance, exosomes from Schwann cells of the PNS have been shown to promote neurite outgrowth in dorsal root ganglia (DRG) neurons, and exosomes from mesenchymal cells enhance neurite outgrowth in DRG and CNS-derived cortical neurons [18,19,20,21]. In the case of fibroblasts, exosomes from these cells can promote neurite outgrowth in DRG neurons in normal, uninjured contexts and can enhance axon regeneration in injured sciatic nerves [103]. In addition, fibroblasts can promote Schwann cell migration and sorting, both of which are associated with axonal regrowth [23,104,105]. In our study, we found that fibroblast-derived sEVs can promote axon outgrowth in cortical neurons and that they act through the Wnt-PCP pathway. In contrast, in a prior study, fibroblast-derived exosomes were shown to promote axon regeneration in a Wnt-dependent manner but did so via the mTOR pathway and only in neurons grown in an inhibitory environment [24]. Thus, exosomes appear to be capable of activating distinct neuronal signaling pathways in growth-permissive versus inhibitory contexts. We showed that the intact sEVs are required to promote axon outgrowth. Therefore, we speculate that fibroblast-derived sEVs may contain surface proteins that are directly involved in axon outgrowth. Alternatively, these surface proteins may be required for EV uptake, with axon outgrowth being induced by sEV luminal proteins. Further investigations are required to explore these possibilities. In contrast to fibroblasts, we observed that sEVs derived from primary neurons or unactivated astrocytes had no effect on neurite outgrowth, even when used at higher concentrations. However, there is prior evidence of neuronal sEVs promoting neurogenesis and providing trophic support to neurons [106,107]. Moreover, and consistent with our findings, sEVs derived from unactivated astrocytes can also promote neuronal survival but exhibit no effect on neurite outgrowth [108]. Upon damage, activated astrocytes contribute to the inhibitory microenvironment by forming a glial scar at the injury site [96,97,98]. In line with this, we observed that sEVs derived from LPS-activated astrocytes inhibit neurite outgrowth, similar to a previous report showing that exosomes from Interleukin-1β-activated human primary astrocytes inhibited neurite outgrowth in mouse cortical neurons [109]. This is consistent with the notion that astrocyte-derived sEVs harbor a stimulus-dependent cargo, which could confer different functional properties to sEVs [13]. For instance, a trophic stimulus to astrocytes induces the secretion of neuroprotective sEVs [110], whereas an inflammatory stimulus causes the production of sEVs that inhibit neurite outgrowth and neuronal activity [109,111]. This suggests that the cell type and state of exosome-secreting cells determine the axon growth-promoting properties of exosomes. Understanding the mechanisms that alter exosome cargos to thereby modulate the effects on neurons is an important area for future investigation.

Core PCP components modulate many aspects of neuronal morphology, including neuronal polarization, axon outgrowth, axon guidance, and synapse formation [31,36,37,38]. Our data show that both of the related PCP components, Pk1 and Pk2, are required for growth of the prospective axon, which is consistent with a recent study showing that Pk2 is necessary for proper neuronal polarity in rat hippocampal neurons [112]. We also observed that the absence of Pk1 and Pk2 attenuated sEV-induced growth of the longest neurite. Another core PCP component, Vangl2, has been reported to exhibit differential effects in distinct neurons. For instance, downregulation of Vangl2 in commissural neurons using shRNAs inhibited Wnt5a-induced axonal outgrowth [85], whereas hippocampal neurons from Vangl2 conditional-knockout mice exhibited increased basal and Wnt5a-induced axon outgrowth [113]. In our study, we observed that both Vangl1 and Vangl2 are required for basal neurite outgrowth and that only Vangl2 is required for sEV-induced neurite outgrowth. Importantly, sEVs induced the relocalization of Vangl2 to the distal part of the axon, further supporting a role for Vangl2 in sEV-mediated axon outgrowth. Although not designated as core PCP components, members of the Smurf E3 ubiquitin ligase family are important regulators of PCP and can modulate neuronal morphology [49,75,76,77]. Consistently, we found that both Smurf1 and Smurf2 are required for neurite outgrowth and that sEV-induced neurite outgrowth is attenuated in Smurf1 and Smurf2 knockout neurons. Taken together, these results demonstrate that sEVs engage key components of the Wnt/PCP pathway to modulate neuritogenesis.

Fzd receptors and Dvls are essential for PCP signaling but also mediate canonical Wnt/β-catenin signals [78]. Diverse Fzd receptors have been shown to modulate events that establish neuronal morphology, including axonal polarity and growth, axon guidance, dendrite morphogenesis, and synaptogenesis [84]. In particular, Fzd3, 5, and 9 have been reported to be essential for axon outgrowth and guidance [84]. In embryonic cortical neurons (E15.5-16.5), we observed that Fzd1, 2, 3, 7, 8, and 9 are expressed at varying levels. Of these Fzds, individual knockdown of Fzd1, 3, or 9 attenuated both basal and sEV-induced neurite outgrowth, whereas Fzd7 was only required for neurite outgrowth mediated by sEVs. This indicates that Fzd1, 3, and 9 act in a non-redundant manner and that Fzd7 is specifically required for sEV-mediated effects. Consistently, a non-redundant role for Fzd3 in mediating axon outgrowth and guidance in knockout mouse models has been described [114,115,116]. The cell surface and intracellular distribution of Fzds can be modulated by post-translational modifications such as phosphorylation [84]. Moreover, in neurons, a distinctive spatiotemporal localization of Fzds in the soma, axon, and growth cone during different developmental stages has been observed [117,118,119]. This differential localization of Fzds in space and time, due at least in part to changes in post-translational modifications, could be critical for axon formation and outgrowth, and thus may explain the non-redundant role of Fzds during this process. Dvls are shared by PCP and canonical Wnt pathways and have been reported to modulate axon outgrowth and guidance [80,81]. For instance, Dvl2 has been reported to specifically function in PCP signaling [120]. We observed that cortical neurons express all three Dvls (Dvl1, 2, and 3), but only Dvl2 was required for sEV-induced neurite outgrowth, a process that we have shown requires PCP signaling. Altogether, our data indicate a key role for the PCP pathway in regulating neuronal polarization and axon outgrowth that is enhanced by fibroblast-derived sEVs.

In vertebrates, Wnts are required for the activation of PCP signaling and can modulate neuronal morphology [26,33,121]. Our data show that neuronal Wnt secretion is required for axon formation, as chemical inhibition of Porcupine or siRNA-mediated knockdown of Wls, both of which block Wnt secretion, prevented axonogenesis. This observation is consistent with a previous report showing that treatment with the Porcupine inhibitor, IWP2, inhibits neuronal polarization in hippocampal neurons [122]. Moreover, we observed that sEV-induced neurite outgrowth was inhibited upon blocking neuronal Wnt secretion. We showed that cortical neurons express Wnt5a, 5b, 7a, and 7b, three of which (Wnt5b, 7a, and 7b) are required for the growth of the prospective axon and for enhanced axon elongation induced by sEVs. Intracellular association of Wnts with exosomes is critical for Wnt secretion on exosomes [40,86,87]. Consistent with this, we showed that neuronal Wnt7b, the most highly expressed Wnt in E15.5 cortical neurons, can colocalize with the exogenously applied CD81-positive sEVs within the neurons. This suggests the possibility that sEVs internalized by neurons can engage endogenous Wnts in the context of neurite outgrowth. In a prior study in cancer cells, we showed that exosomes from fibroblasts acquire Wnt11 when internalized by breast cancer cells to activate autocrine Wnt-PCP signaling and promote cell motility and formation of protrusions [25]. Thus, we speculate that sEVs might similarly activate autocrine Wnt-PCP signaling in neurons to promote axon outgrowth, though precisely how remains to be explored.

Although the fibroblasts used in our study were derived from skin, lung, and mouse embryos, and thus do not reside in the CNS, the current study identifies the potential of fibroblast-derived sEVs, irrespective of the tissue origin, in activating axon outgrowth systems. Whether this might be useful as a therapeutic for neuronal injury would be worth further exploration. The role of CNS fibroblasts in development, maintenance, and CNS diseases is not well understood but is beginning to be explored [123]. Thus, studying the effect of sEVs from CNS fibroblasts, such as from the meninges, choroid plexus, and perivascular spaces on axon outgrowth, will also be an important avenue for future exploration. Other resident cells of the CNS, including oligodendrocytes and microglia, have also been reported to modulate neuronal functions through sEVs [13]. Whether and how these sEVs affect axon outgrowth needs to be explored further. At the molecular level, we have shown that sEVs induce axon elongation and a polarized neuronal morphology by engaging Wnt-PCP signaling components. As context-dependent effects of exosomes are not well understood, it will be critical to explore the molecular determinants in exosomes that confer context specificity in varying biological functions.

## Figures and Tables

**Table 1:** List of primers for genotyping.

Gene	Primers
*Pk1-F*	CAGACTCCTGTCAACGCGATG
*Pk1-R*	CGCCTGCACAACAGCACTGTC
*Pk1-MT-R*	CCCCACAACGGGTTCTTCTGTTAG
*Pk2-F*	TGTGGCCACATTTGTTTTAGAGG
*Pk2-R*	CACACACTAACCAGAAATATCATC
*Pk2-MT-R*	CCCCACAACGGGTTCTTCTGTTAG
*Vangl2-F*	TGGCTGTCTTCTGCACTCAC
*Vangl2-R*	GCACCTTCTTGGTGCTCACT
*Smurf1-F*	CAGATGCTGAGGCAGGAGAATCATGAG
*Smurf1-R*	CAGTCCCCTCCCAACACACAATCAC
*Smurf1-MT-R*	AGTTCTAATTCCATCAGAAGCTGACTCTAG
*Smurf2-F*	TGCTATGGCTTCTGACTATTAAAAGGTC
*Smurf2-R*	CTTAAACATCAGCTCCTGAGTACACAAC
*Smurf2-MT-R*	AGTTCTAATTCCATCAGAAGCTGACTCTAG
*Cre-F*	TGACCGTACACCAAAATTTG
*Cre-R*	ATTGCCCCTGTTTCACTATC

**Table 3:** List of siRNAs.

Gene	Dharmacon Catalog Number
*Dvl1*	D-040910-01, D-040910-02, D-040910-03, D-040910-04
*Dvl2*	D-040921-01, D-040921-02, D-040921-03, D-040921-04
*Dvl3*	D-040922-01, D-040922-02, D-040922-03, D-040922-04
*Frizzled1*	D-063230-01, D-063230-02, D-063230-03, D-063230-04
*Frizzled2*	D-040443-01, D-040443-02, D-040443-03, D-040443-04
*Frizzled3*	D-048416-01, D-048416-02, D-048416-03, D-048416-04
*Frizzled7*	D-041631-01, D-041631-02, D-041631-03, D-041631-04
*Frizzled8*	D-045544-01, D-045544-02, D-045544-03, D-045544-04
*Frizzled9*	D-040416-01, D-040416-02, D-040416-03, D-040416-04
*Prickle1*	D-042729-01, D-042729-02, D-042729-03, D-042729-04
*Prickle2*	D-056882-01, D-056882-02, D-056882-05, D-056882-06
*Vangl1*	D-057276-01, D-057276-02, D-057276-03, D-057276-04
*Vangl2*	D-059396-01, D-059396-02, D-059396-03, D-059396-17
*Wnt5a*	D-065584-01, D-065584-02, D-065584-03, D-065584-04
*Wnt5b*	D-060428-01, D-060428-03, D-060428-04, D-060428-17
*Wnt7a*	D-058932-02, D-058932-03
*Wnt7b*	D-042139-01, D-042139-02, D-042139-03, D-042139-04
*Porcupine*	D-049203-01, D-049203-02, D-049203-03, D-049203-04
*Wntless*	D-060922-01, D-060922-02, D-060922-03, D-060922-04

**Table 4:** List of qPCR primers.

Gene	Forward Primer	Reverse Primer
*Wnt3a*	CTCCTCTCGGATACCTCTTAGTG	GCATGATCTCCACGTAGTTCCTG
*Wnt5a*	CAACTGGCAGGACTTTCTCAA	CATCTCCGATGCCGGAACT
*Wnt5b*	CTGCTGACTGACGCCAACT	CCTGATACAACTGACACAGCTTT
*Wnt7a*	GGTGCGAGCATCATCTGTAA	CATTTGGGAGCCTTCTCCTATG
*Wnt7b*	GGATGCCCGTGAGATCAAA	GACACACCGTGACACTTACA
*Prickle1*	TCCCGAAACAAGGTCAGATTTA	TCTCTGGATCTGGCTGACT
*Prickle2*	CACTGCTTTGAGTCCCTGTATG	TCTGTAGCATGCCAGTGTTG
*Vangl1*	GCTGGCCTGAAAGTCTACAA	CGTGTTCGGCCTCTTCATAATA
*Vangl2*	ACTCGGGCTATTCCTACAAGT	TGATTTATCTCCACGACTCCCAT
*Frizzled1*	GCTGAGCTTGGAACTTTGTG	AGCCTCCAGCAACCAAA
*Frizzled2*	GCCGTCCTATCTCAGCTATAAGT	TCTCCTCTTGCGAGAAGAACATA
*Frizzled3*	ATGGCTGTGAGCTGGATTGTC	GGCACATCCTCAAGGTTATAGGT
*Frizzled7*	ATATCACGGCGAGAAAGGC	GCAGGATGGTCTGGTTGTAG
*Frizzled8*	ATGGAGTGGGGTTACCTGTTG	CACCGTGATCTCTTGGCAC
*Frizzled9*	TCCGCGTTGTGTTTCTTCT	GGCCAAGGAGTAGACATTGTAG
*Dvl1*	GAAGTCAGGCTTCAGCTGTT	CTACCTGTAAGTTCTGGAGGGA
*Dvl2*	CCATCCTTCCACCCTAATGTATC	CCATGTTCACTGCTGTCTCT
*Dvl3*	TGGTTTCATCCGCCATACC	ATCATGGTCGTGAAGCGATAG
*Cd81*	GTACCTGGAACTGGGAAACAA	CCCAGGAAGCCTACAAACAT
*Smpd2*	CAAAGGCTATCGCTCACCTATC	CTGGATTGGGTGTTTGGAGAA
*Wntless*	TGGGAAGCAGTCTAGCCTCC	GCAGCACAAGCCAAGGTGATA
*Porcupine*	GAGAAGGACCACCTGGAATG	ATAAGACATGGGCAGGTTCC
*iNOS2*	GGCAGCCTGTGAGACCTTTG	GCATTGGAAGTGAAGCGTTTC
*IL1b*	CAGGCAGGCAGTATCACTCA	TGTCCTCATCCTGGAAGGTC
*IL6*	ATGGATGCTACCAAACTGGAT	TGAAGGACTCTGGCTTTGTCT
*TNF-* *α*	ACGGCATGGATCTCAAAGAC	GTGGGTGAGGAGCACGTAGT
*Gapdh*	AGGTCGGTGTGAACGGATTTG	TGTAGACCATGTAGTTGAGGTCA

## Data Availability

The data presented in this study are available in the article and Supplementary Materials.

## Supplementary Materials

The following supporting information can be downloaded at: https://www.mdpi.com/article/10.3390/cells14010056/s1, Figure S1: sEVs promote the growth of the longest neurite in a concentration-dependent manner. Related to Figure 1. Figure S2: sEVs promote axon outgrowth and polarized neuronal morphology. Related to Figure 1. Figure S3: sEVs isolated using a discontinuous density gradient promote the growth of the longest neurite. Related to Figure 1. Figure S4: sEVs isolated using size exclusion chromatography promote the growth of the longest neurite. Related to Figure 1. Figure S5: The PCP components, Pk1/2 and Vangl2, are required for sEV-induced growth of the longest neurite. Related to Figure 2. Figure S6: The PCP components, Fzds, are required for sEV-induced growth of the longest neurite. Related to Figure 3. Figure S7: Related to Figure 4 and Figure 5. Figure S8: Related to Figure 5 and Figure 6. Figure S9: Related to Figure 7.
