# Supplementary material for: Small Extracellular Vesicles Promote Axon Outgrowth by Engaging the Wnt-Planar Cell Polarity Pathway
Source: Cells. 2025 Jan 6;14(1):56. doi: 10.3390/cells14010056 (PMC11720052; doi:10.3390/cells14010056)
Supplement: Supplementary file 1 [file cells-14-00056-s001.zip › cells-3295706-supplementary.pdf]

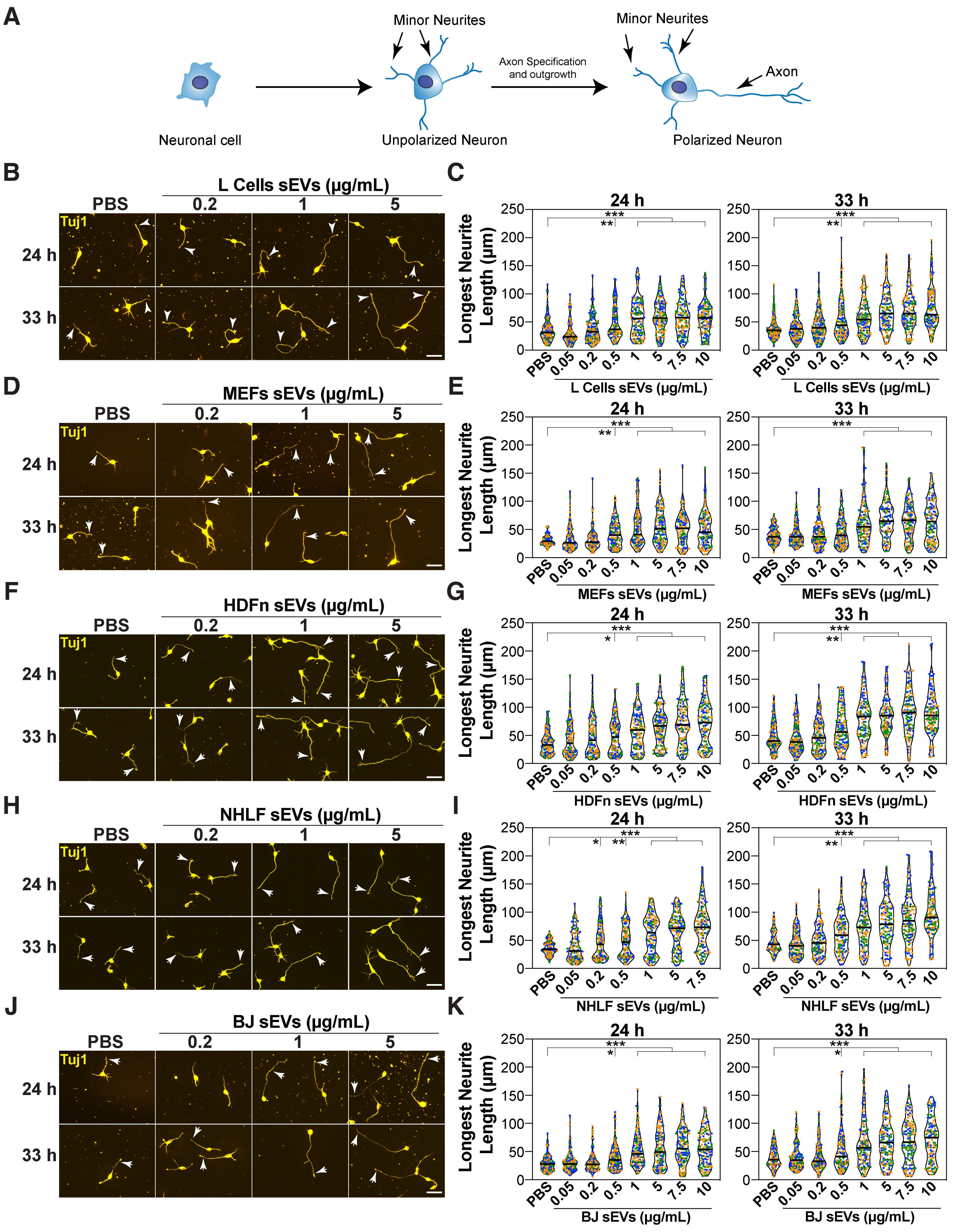
A
Minor Neurites
Minor Neurites
Axon Specification and outgrowth
Axon
Neuronal cell
Unpolarized Neuron
Polarized Neuron
B
L Cells sEVs (µg/mL)
PBS
0.2
1
5
Tuj1
24 h
33 h
C
24 h
33 h
Longest Neurite Length (µm)
L Cells sEVs (µg/mL)
D
MEFs sEVs (µg/mL)
E
MEFs sEVs (µg/mL)
F
HDFn sEVs (µg/mL)
G
HDFn sEVs (µg/mL)
H
NHLF sEVs (µg/mL)
I
NHLF sEVs (µg/mL)
J
BJ sEVs (µg/mL)
K
BJ sEVs (µg/mL)
PBS
0.05
0.2
0.5
1
5
7.5
10

Figure S1

Figure S1. sEVs promote the growth of the longest neurite in a concentration-dependent manner. Related to Fig. 1. (A) A schematic of the morphological changes in neurons cultured *in vitro*. **(B-K)** Dissociated E15.5-16.5 mouse cortical neurons were treated with various concentrations (0.05-10 μg/mL) of sEVs purified from the indicated fibroblast cell lines including L cells **(B, C)**, MEFs **(D, E)**, HDFn **(F, G)**, NHLF **(H, I)** and BJ **(J, K),** 4 h after plating. Neurons were fixed at 24 and 33 h, and neuronal morphology was examined in Tuj1 stained neurons. Representative images **(B, D, F, H, J)** and quantifications **(C, E, G, I, K)** are shown. Arrowheads mark the longest neurite. Scale bar, 40 μm.

Neurite lengths are quantified from a minimum of 90 neurons per condition from 3 independent experiments and plotted as a violin plot with values from each experiment distinctly colored and the median marked by a black line. Statistical significance: *$p<0.05$, **$p<0.01$, ***$p<0.001$ using one-way ANOVA with Dunnett's post-test.

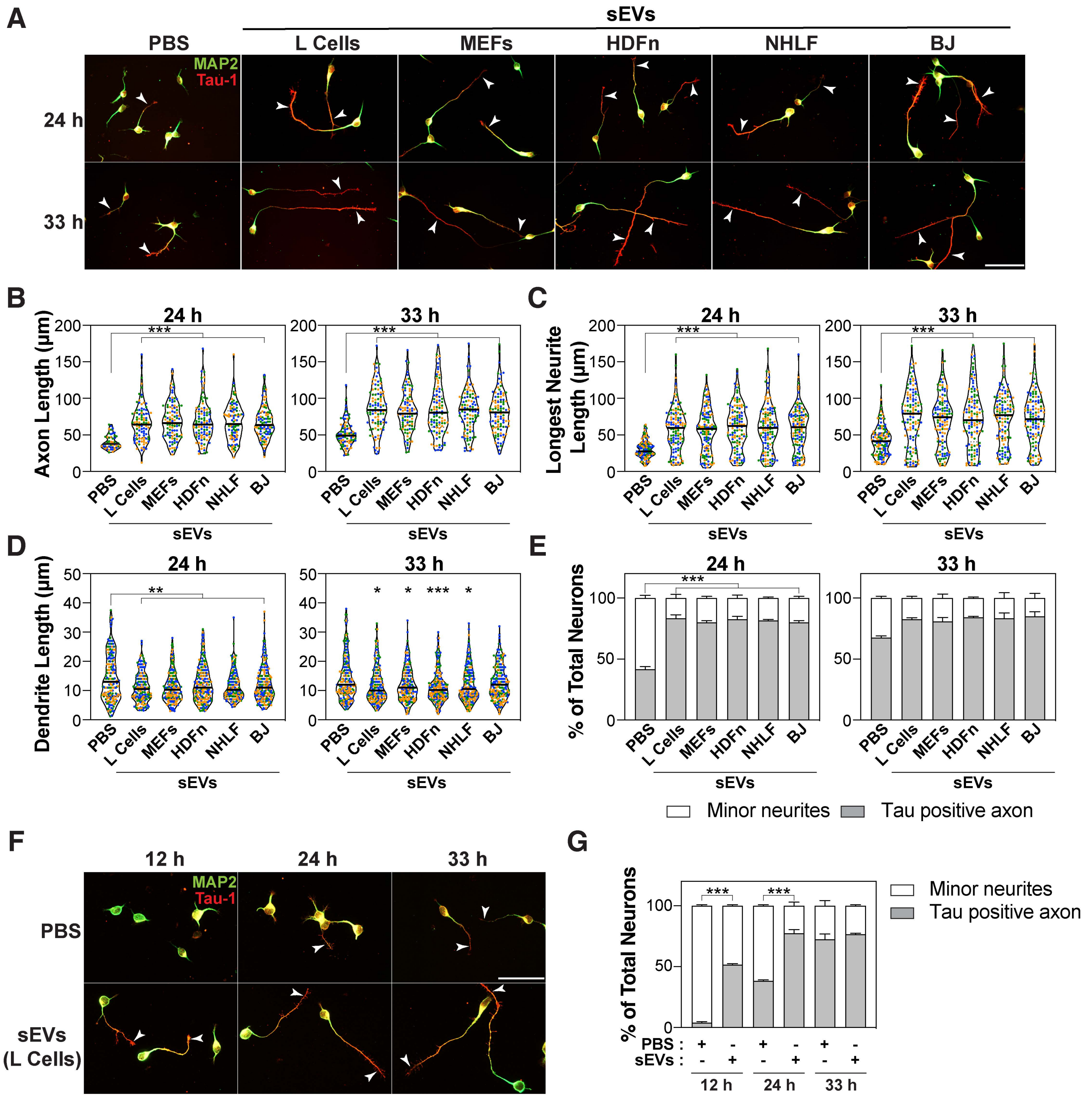
A
sEVs
PBS
L Cells
MEFs
HDFn
NHLF
BJ
MAP2
Tau-1
24 h
33 h
B
Axon Length (µm)
24 h
33 h
C
Longest Neurite Length (µm)
D
Dendrite Length (µm)
E
% of Total Neurons
Minor neurites
Tau positive axon
F
12 h
PBS
sEVs (L Cells)
G
PBS :
sEVs :

Figure S2

Figure S2. sEVs promote axon outgrowth and polarized neuronal morphology. Related to Fig. 1. **(A-G)** Dissociated E15.5-16.5 mouse cortical neurons were treated with sEVs purified from the indicated fibroblast cell lines **(A-E)** or only L cells **(F-G)**, 4 h after plating. Neurons were fixed at 24 and 33 h **(A-E)** or 12, 24 and 33 h **(F-G)**, and neuronal morphology was examined in neurons immunostained for MAP2 (dendrites, green) and Tau-1 (axons, red). **(A, F)** Representative images are shown. Arrowheads mark Tau-1 positive axons. Scale bar, 50 μm. (**B-E**) The length of the Tau-1 positive axons **(B)**, longest neurite (independent of Tau-1+ staining, **C**) and individual dendrite length (MAP2 positive, **D**) is quantified. (**E, G)** Percent of neurons containing only minor neurites (MAP2 positive) or a single Tau-1-positive axon, is plotted.

Neurite lengths are quantified from a minimum of 45 (for 24 h in panel **B**) or 90 (for all others) neurons per condition from 3 independent experiments and plotted as a violin plot with values from each experiment distinctly colored and the median marked by a black line. In panel **B**, for the 24 h control group (PBS treated), less data points were available due to greater percentage of neurons lacking a Tau-1 positive axon. Statistical significance: *p<0.05, **p<0.01, ***p<0.001 using one-way ANOVA with Dunnett's post-test **(B, C, D)**, or two-way ANOVA with Tukey's post-test **(E, G)**.

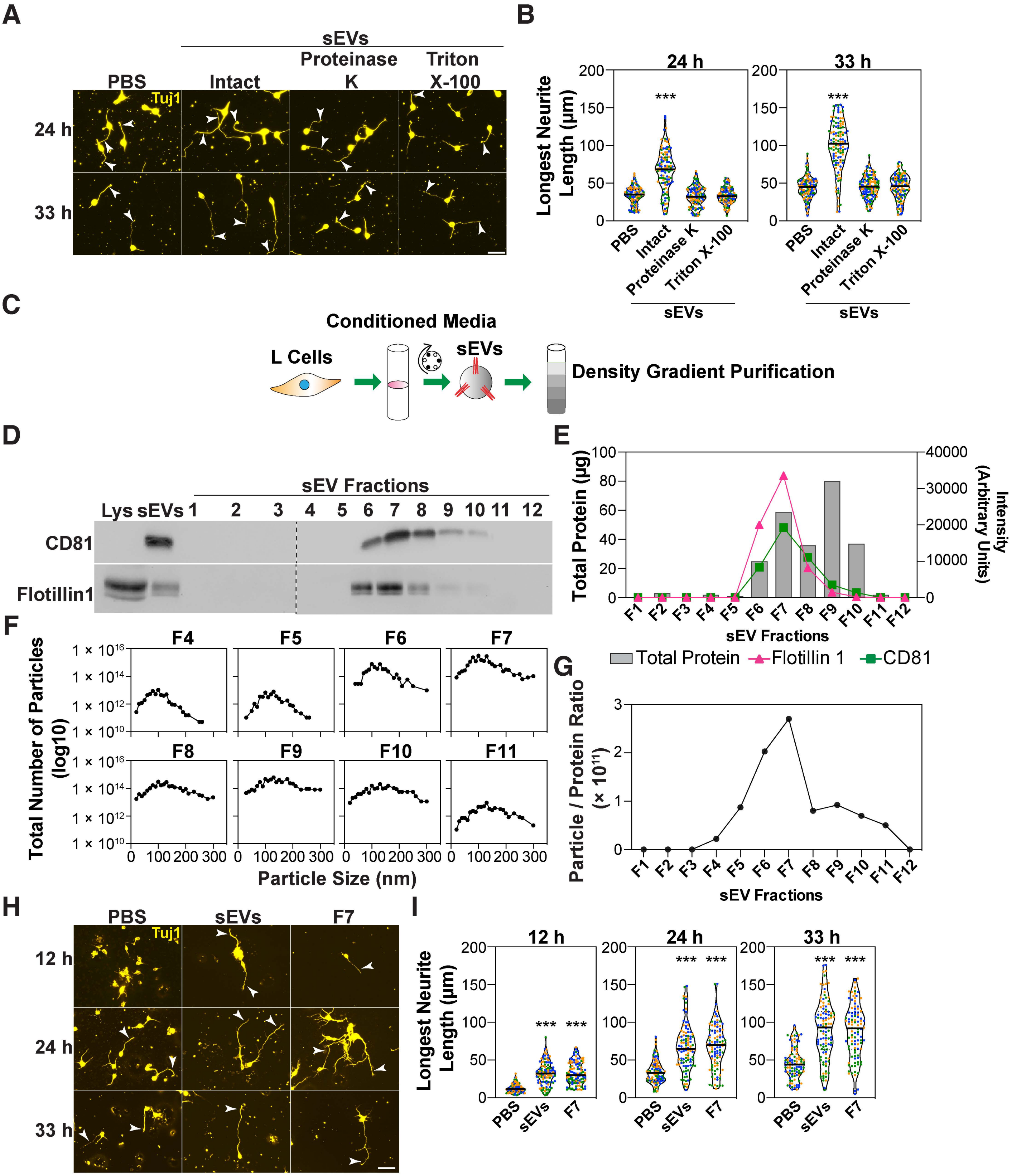

A
B
C
D
E
F
G
H
I
sEVs
PBS
Intact
Proteinase K
Triton X-100
Tuj1
24 h
33 h
Longest Neurite Length (μm)
Conditioned Media
L Cells
Density Gradient Purification
sEV Fractions
Lys
CD81
Flotillin1
Total Protein (μg)
Intensity (Arbitrary Units)
Total Protein
Flotillin 1
CD81
Total Number of Particles (log10)
Particle Size (nm)
Particle / Protein Ratio (× 10¹¹)
F7
12 h

Figure S3

Figure S3. sEVs isolated using a discontinuous density gradient promote the growth of the longest neurite. Related to Fig. 1. (A-B, H-I) Dissociated E15.5-16.5 cortical neurons were treated with the intact sEVs, Proteinase K treated and Triton X-100 treated sEV pellet **(A-B)** or sEV pellet and the F7 fraction (both at 2×10^8 particles/mL) **(H-I)**, 4 h after plating. Neuronal morphology was examined in Tuj1 stained neurons at 24 and 33 h **(A-B)** or 12, 24 and 33 h **(H-I)**. Representative images **(A, H)** and quantifications **(B, I)** are shown. Arrowheads mark the longest neurite. Scale bar, 40 µm. **(C)** A schematic of the experimental set up. sEV pellets (100,000 x g) purified from the conditioned media (CM) of L cells using differential centrifugation were subjected to iodixanol discontinuous density gradient purification. (**D)** Twelve fractions were collected starting from the top of the discontinuous gradient and were analyzed by immunoblotting for the indicated EV markers. (**E)** Intensity of the bands of EV markers from **(D)** is plotted against the protein concentration. (**F)** Particle size distribution was determined using NTA. (**G)** Fraction 7 (F7) has the highest particle number/protein ratio.
Neurite lengths are quantified from a minimum of 90 neurons per condition from 3 independent experiments and plotted as a violin plot with values from each experiment distinctly colored and the median marked by a black line. Statistical significance: ***$p<0.001$ using one-way ANOVA with Dunnett's post-test.

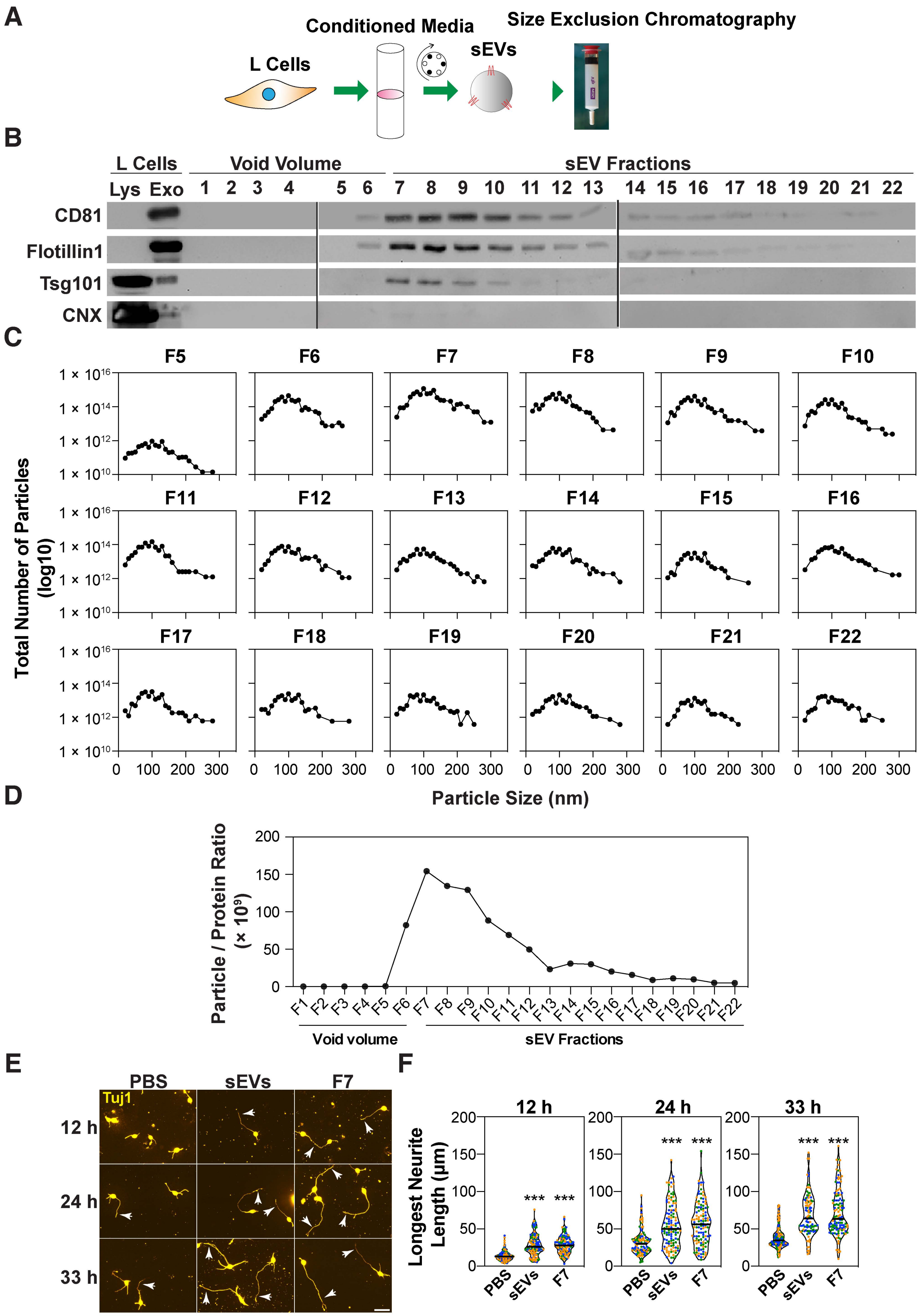
A
L Cells
Conditioned Media
sEVs
Size Exclusion Chromatography
B
L Cells
Lys Exo
Void Volume
sEV Fractions
CD81
Flotillin1
Tsg101
CNX
C
Total Number of Particles (log10)
Particle Size (nm)
D
Particle / Protein Ratio (× 10⁹)
Void volume
sEV Fractions
E
PBS
sEVs
F7
Tuj1
12 h
24 h
33 h
F
Longest Neurite Length (μm)
12 h
24 h
33 h
PBS
sEVs
F7
***

Figure S4

Figure S4. sEVs isolated using size exclusion chromatography promote the growth of the longest neurite. Related to Fig. 1. **(A)** A schematic illustration of experimental setup. sEVs purified from the CM of L cells were subjected to size exclusion chromatography. **(B)** A total of 22 fractions were collected and subjected to immunoblotting for EV markers. (**C)** Particle size was measured using NTA. **(D)** Fraction 7 (F7) has the highest particle number/protein ratio. **(E)** F7 fraction promotes the growth of the longest neurite. Dissociated E15.5-16.5 cortical neurons were treated with resuspended sEV pellet (5 μg/mL) and F7 fraction (5 μg/mL), 4 h after plating. Neuronal morphology was examined in Tuj1 stained neurons at various time points: 12, 24 and 33 h. Representative images are shown. Arrowheads mark the longest neurite. Scale bar, 40 μm. **(F)** The length of the longest neurite was quantified from a minimum of 90 neurons per condition from 3 independent experiments and plotted as a violin plot with values from each experiment distinctly colored and the median marked by a black line. Statistical significance: ***$p<0.001$ using one-way ANOVA with Dunnett's post-test.

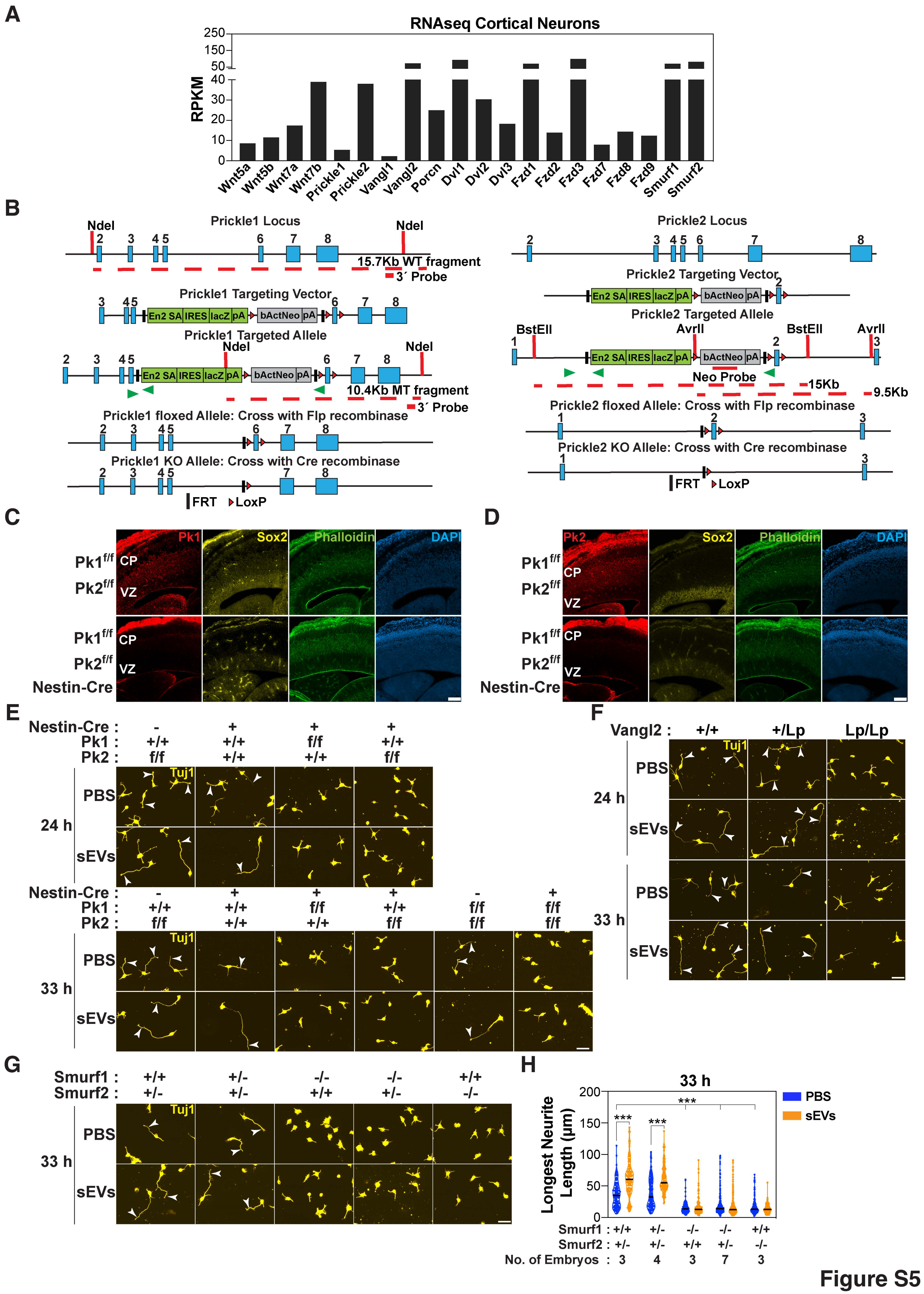
A
RNAseq Cortical Neurons
RPKM
Wnt5a
Wnt5b
Wnt7a
Wnt7b
Prickle1
Prickle2
Vangl1
Vangl2
Porcn
Dvl1
Dvl2
Dvl3
Fzd1
Fzd2
Fzd3
Fzd7
Fzd8
Fzd9
Smurf1
Smurf2
B
Prickle1 Locus
NdeI
15.7Kb WT fragment
3´ Probe
Prickle1 Targeting Vector
En2 SA IRES lacZ pA
bActNeo pA
Prickle1 Targeted Allele
10.4Kb MT fragment
Prickle1 floxed Allele: Cross with Flp recombinase
Prickle1 KO Allele: Cross with Cre recombinase
FRT
LoxP
Prickle2 Locus
Prickle2 Targeting Vector
Prickle2 Targeted Allele
BstEII
AvrII
Neo Probe
15Kb
9.5Kb
Prickle2 floxed Allele: Cross with Flp recombinase
Prickle2 KO Allele: Cross with Cre recombinase
C
Pk1
Sox2
Phalloidin
DAPI
CP
VZ
Nestin-Cre
D
Pk2
E
Nestin-Cre :
Pk1 :
Pk2 :
Tuj1
PBS
sEVs
24 h
33 h
F
Vangl2 :
+/+
+/Lp
Lp/Lp
G
Smurf1 :
Smurf2 :
H
33 h
Longest Neurite Length (μm)
PBS
sEVs
No. of Embryos :
Figure S5

Figure S5. The PCP components, Pk1/2 and Vangl2, are required for sEV-induced growth of the longest neurite. Related to Fig. 2. (A) Expression of PCP genes and Wnts in cortical neurons. Total RNA was isolated from E15.5-16.5 cortical neurons after 33 h of *in vitro* culturing. An RNA sequence library was constructed, and high-quality reads were aligned to the *M. musculus* genome and expression of the indicated genes plotted. RPKM, Reads per kilobase of transcript per million mapped reads. (**B)** Schematic maps of the Pk1 and Pk2 wild type allele, targeting vector, targeted allele, floxed allele after crossing with Flp recombinase and knockout allele after crossing with Cre recombinase are shown. Green arrowheads indicate the position of PCR primers for genotyping. **(C, D)** Brain cryosections of E15.5 mouse embryo were stained with antibodies for Pk1 and Sox2 **(C)** or Pk2 and Sox2 **(D)** along with phalloidin. CP: cortical plate, VZ: ventricular zone. Scale bar, 100 µm. **(E-H)** Cortical neurons (E15.5-16.5) were isolated from Pk1 and Pk2 conditional knockout mice **(E)** or Vangl2 mutant littermates **(F)** or Smurf1 and Smurf2 knockout mice **(G, H)**, and treated with sEVs from L cells, 4 h after plating. Neurons were fixed at 24 and 33 h and stained with Tuj1. Representative images are shown. Arrowheads mark the longest neurite. Scale bar, 40 µm. **(H)** The length of the longest neurite was quantified from 30 neurons per embryo, and the total number of embryos analyzed was indicated below the genotypes. Statistical significance: ***$p<0.001$ using two-way ANOVA with Tukey's post-test.

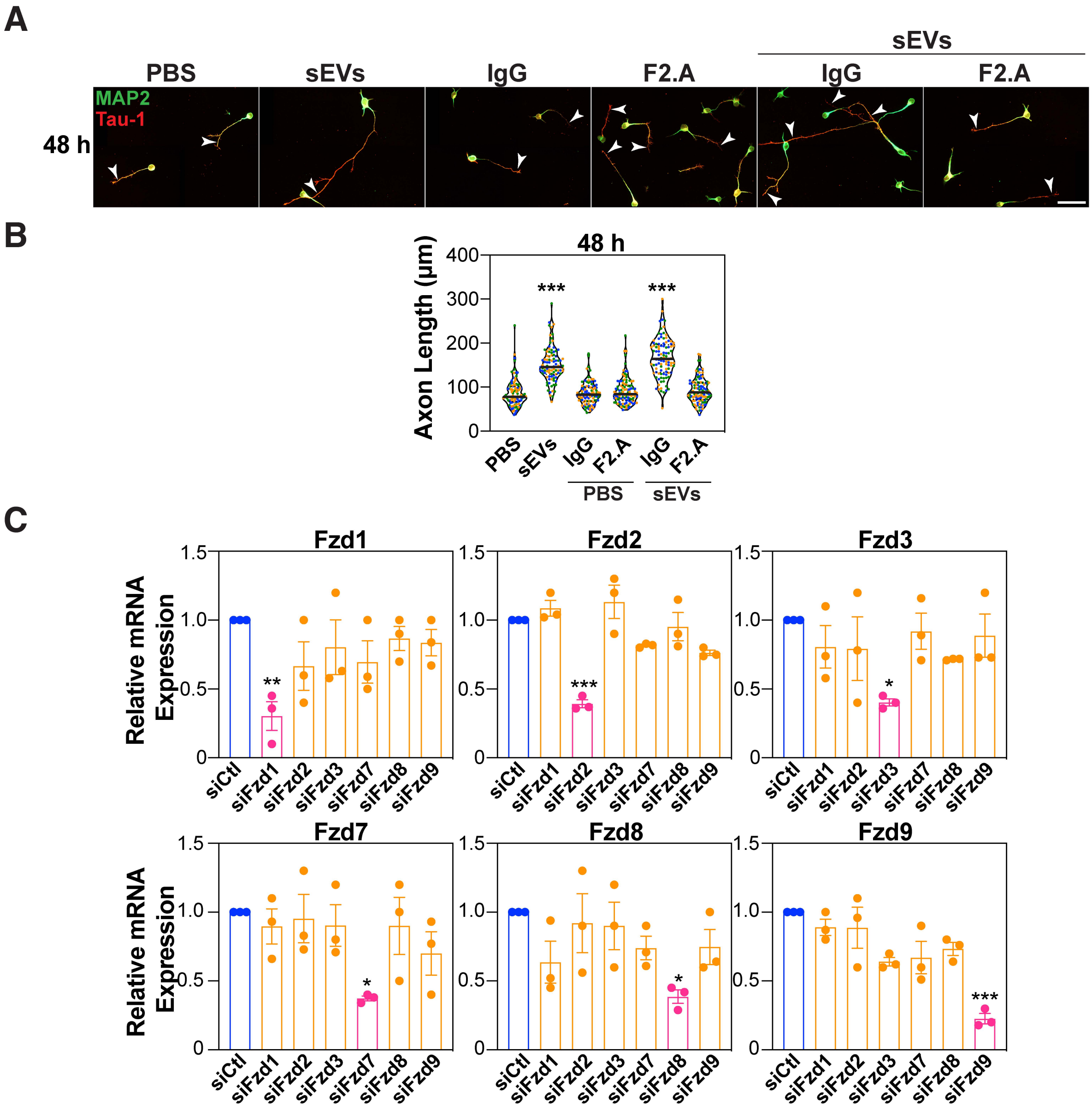
A
PBS
sEVs
IgG
F2.A
sEVs
IgG
F2.A
MAP2
Tau-1
48 h
B
48 h
Axon Length (μm)
***
***
PBS
sEVs
IgG
F2.A
IgG
F2.A
PBS
sEVs
C
Fzd1
Fzd2
Fzd3
Fzd7
Fzd8
Fzd9
Relative mRNA Expression
siCtl
siFzd1
siFzd2
siFzd3
siFzd7
siFzd8
siFzd9

Figure S6

Figure S6. The PCP components, Fzds, are required for sEV-induced growth of the longest neurite. Related to Fig. 3. (A-B) Dissociated E15.5-16.5 mouse cortical neurons were treated with sEVs (5 µg/mL) from L cells and IgG or a Fzd blocking antibody, F2.A (100 nM), 24 h after plating. Neurons were fixed at 48 h, and neuronal morphology was examined in neurons immunostained for MAP2 (dendrites, green) and Tau-1 (axons, red). Representative images are shown **(A)**. Arrowheads mark Tau-1 positive axons. Scale bar, 50 µm. **(B)** The length of the Tau-1 positive axons was quantified from a minimum of 90 neurons per condition from 3 independent experiments and plotted as a violin plot with values from each experiment distinctly colored and the median marked by a black line. **(C)** Knockdown efficiency for Fzds was determined in GFP-positive neurons isolated by FACS. Relative mRNA expression was determined by qPCR and is plotted as the mean ± SEM from 3 independent experiments. Statistical significance: *p<0.05, **p<0.01, ***p<0.001 using one-way ANOVA with Dunnett's post-test.

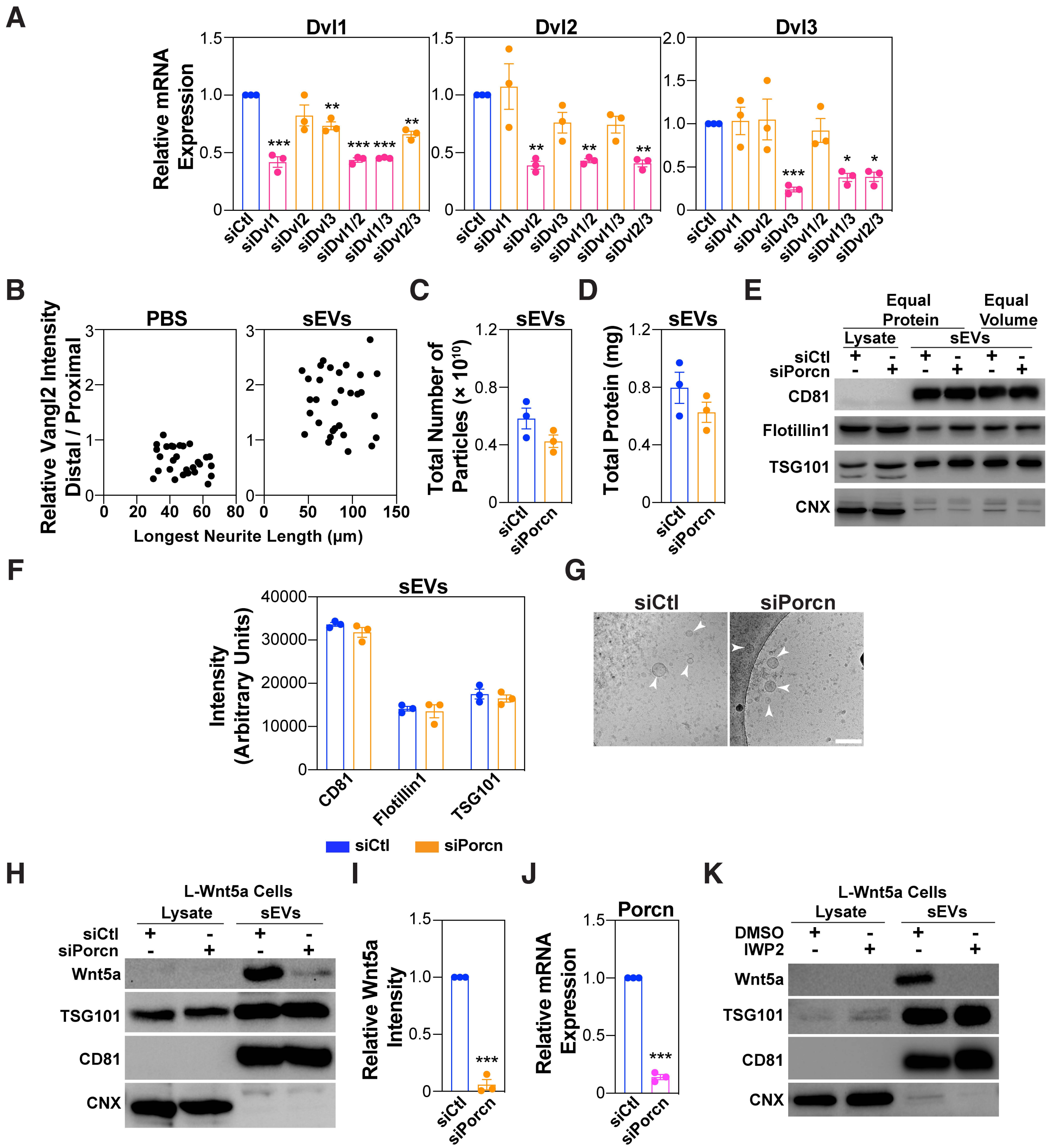
A
Dvl1
Dvl2
Dvl3
Relative mRNA Expression
siCtl siDvl1 siDvl2 siDvl3 siDvl1/2 siDvl1/3 siDvl2/3
B
PBS
sEVs
Relative Vangl2 Intensity Distal / Proximal
Longest Neurite Length (μm)
C
sEVs
Total Number of Particles (× 10¹⁰)
D
sEVs
Total Protein (mg)
E
Equal Protein
Equal Volume
Lysate
sEVs
siCtl
siPorcn
CD81
Flotillin1
TSG101
CNX
F
sEVs
Intensity (Arbitrary Units)
CD81
Flotillin1
TSG101
siCtl
siPorcn
G
siCtl
siPorcn
H
L-Wnt5a Cells
Lysate
sEVs
siCtl
siPorcn
Wnt5a
TSG101
CD81
CNX
I
Relative Wnt5a Intensity
J
Porcn
Relative mRNA Expression
K
L-Wnt5a Cells
Lysate
sEVs
DMSO
IWP2
Wnt5a
TSG101
CD81
CNX

Figure S7

Figure S7. Related to Fig. 4 and 5. (A) Knockdown efficiency for Dvls was determined in GFP-positive neurons isolated by FACS. Relative mRNA expression was determined by qPCR and is plotted as the mean ± SEM from 3 independent experiments. (**B)** The ratio of distal/proximal Vangl2 intensity as a function of the longest neurite length in neurons treated with PBS or sEVs is quantified from 30 neurons from 3 independent experiments. (**C)** The total number of particles in the sEV pellet (100,000 x g) were measured using NTA. (**D)** The total amount of protein in the sEV pellet was measured by Bradford assay. (**E)** Characterization of sEVs derived from porcupine-deficient L cells. A representative immunoblot of the cell lysate and 100,000 x g pellet (sEVs) analyzed for EV markers and calnexin (CNX). (**F)** Quantification of **(E)**. sEVs were collected from an equal number of cells, and equal volumes of sEV pellets (right two lanes) were used for comparison. Protein bands were quantified using ImageJ. **(G)** Representative TEM images of sEV pellet. Arrowheads indicate round vesicles. Scale bar, 200 nm. **(H-K)** Porcupine inhibition blocks Wnt5a secretion in sEVs. L-Wnt5a cells were treated with siCtl or siPorcupine (siPorcn) **(H-J)** and DMSO or IWP2 (10 μM) **(K)** followed by isolation of sEVs. A representative immunoblot of sEVs and lysates indicating the level of Wnt5a, EV markers and CNX is shown **(H, K)**. **(I)** Quantification of **(H)**. Protein bands were quantified using ImageJ. **(J)** Knockdown efficiency of Porcupine in L-Wnt5a cells.
Values are plotted as the mean ± SEM from 3 independent experiments **(A, C, D, F, I, J)**. Statistical significance: *p<0.05, **p<0.01, ***p<0.001 using unpaired t-test **(C, D, I, J)**, one-way ANOVA with Dunnett's post-test **(A)**, or two-way ANOVA with Tukey's post-test **(F)**.

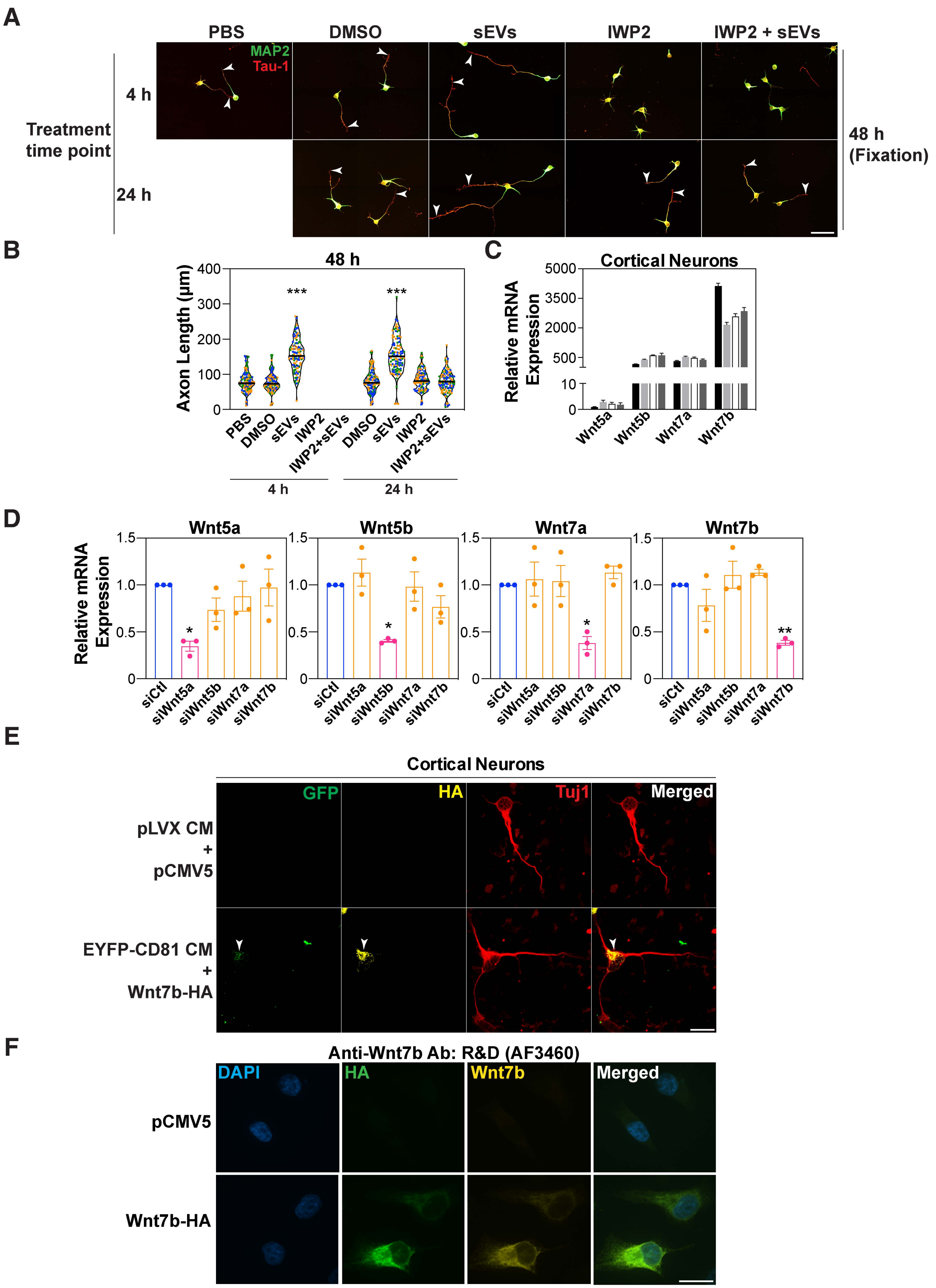
A
PBS
DMSO
sEVs
IWP2
IWP2 + sEVs
MAP2
Tau-1
Treatment time point
4 h
24 h
48 h (Fixation)
B
48 h
Axon Length (μm)
***
***
PBS
DMSO
sEVs
IWP2
IWP2+sEVs
4 h
24 h
C
Cortical Neurons
Relative mRNA Expression
Wnt5a
Wnt5b
Wnt7a
Wnt7b
D
Wnt5a
Wnt5b
Wnt7a
Wnt7b
Relative mRNA Expression
siCtl
siWnt5a
siWnt5b
siWnt7a
siWnt7b
E
Cortical Neurons
GFP
HA
Tuj1
Merged
pLVX CM + pCMV5
EYFP-CD81 CM + Wnt7b-HA
F
Anti-Wnt7b Ab: R&D (AF3460)
DAPI
HA
Wnt7b
Merged
pCMV5
Wnt7b-HA

Figure S8

Figure S8. Related to Fig. 5 and 6. (A-B) Dissociated E15.5-16.5 mouse cortical neurons were treated with sEVs (5 μg/mL) from L cells and DMSO or IWP2 (10 μM), 4 h or 24 h after plating. Neurons were fixed at 48 h, and neuronal morphology was examined in neurons immunostained for MAP2 (dendrites, green) and Tau-1 (axons, red). Representative images are shown **(A)**. Arrowheads mark Tau-1 positive axons. Scale bar, 50 μm. **(B)** The length of the Tau-1 positive axons was quantified from a minimum of 90 neurons per condition from 3 independent experiments and plotted as a violin plot with values from each experiment distinctly colored and the median marked by a black line. For IWP2-treated neurons at 4 h, a comparable number of data points were not available due to lack of Tau-1 positive axons. **(C)** Expression of Wnts in cortical neurons. RNA was isolated from cortical neurons at 0, 12, 24 and 33 h of culturing. A representative plot indicating mRNA expression relative to Wnt5a, determined by qPCR from 2 independent experiments is shown. **(D)** Efficiency of siRNA-mediated knockdown of Wnts. RNA was isolated from GFP-positive neurons after FACS. Relative mRNA expression was determined by qPCR. **(E)** Overlap of the localization of sEVs and Wnt7b-HA. Cortical neurons electroporated with 2 μg of either pCMV5 or C-terminal HA-tagged Wnt7b (Wnt7b-HA) were treated with 10X CM containing CD81-EYFP, 24 h after plating for 30 min. Representative images of neurons stained with GFP (green), HA (yellow) and Tuj1 (red) antibodies from 20 neurons from 2 independent experiments are shown. Arrowheads indicate the localization of Wnt7b and sEVs. Scale bar, 20 μm. **(F)** Wnt7b antibody characterization. MDA-MB-231 cells were transfected with 2 μg of either pCMV5 control or C-terminal HA-tagged Wnt7b (Wnt7b-HA). Cells were fixed after 48 h and immunostained with HA (green) and Wnt7b (yellow) antibodies. Scale bar, 20 μm.
*$p<0.05$, **$p<0.01$, ***$p<0.001$ using one-way ANOVA with Dunnett's post-test **(B, D)**.

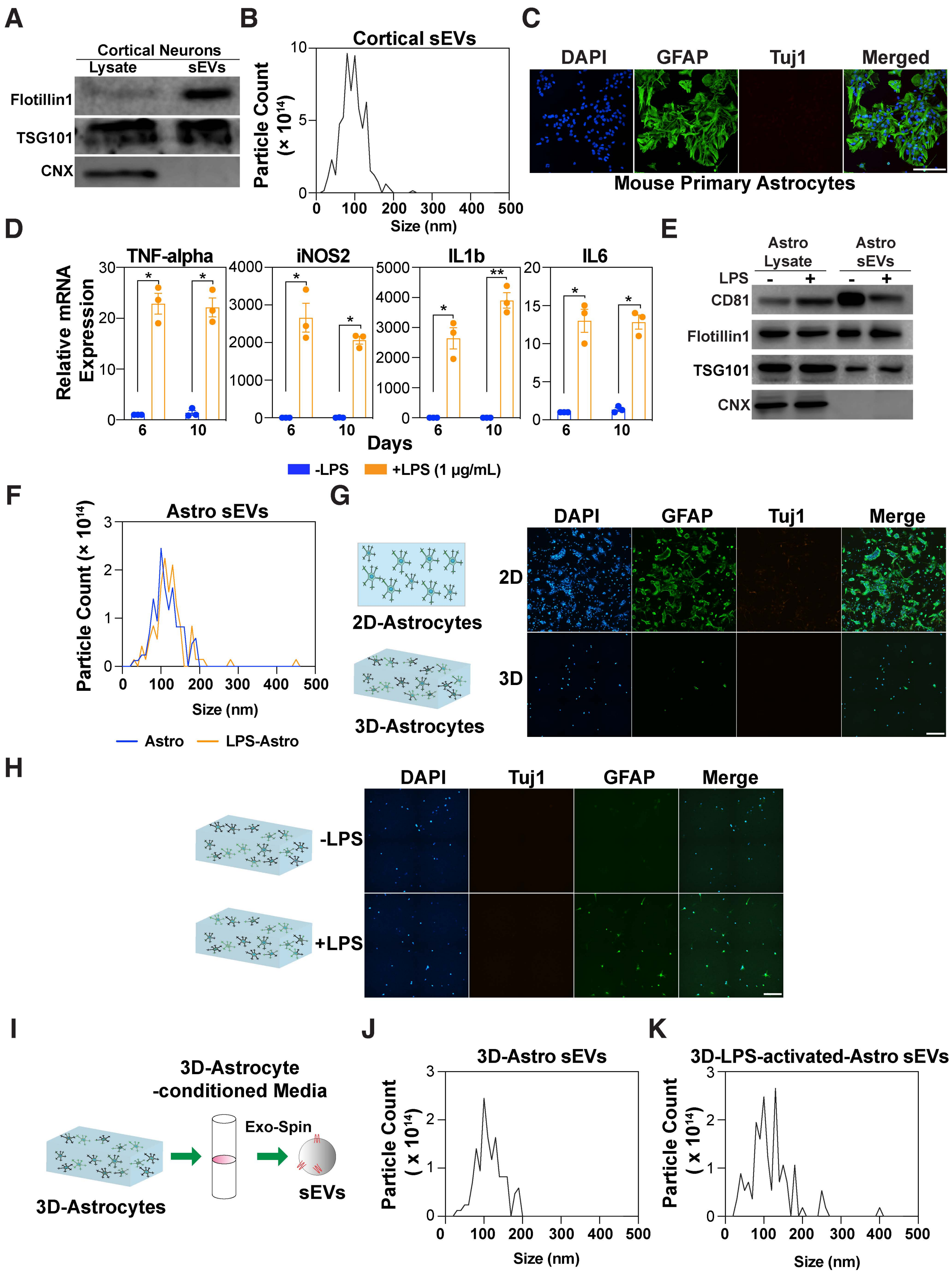
A
Cortical Neurons
Lysate
sEVs
Flotillin1
TSG101
CNX
B
Cortical sEVs
Particle Count (× 10^14)
Size (nm)
C
DAPI
GFAP
Tuj1
Merged
Mouse Primary Astrocytes
D
TNF-alpha
iNOS2
IL1b
IL6
Relative mRNA Expression
Days
-LPS
+LPS (1 μg/mL)
E
Astro Lysate
Astro sEVs
LPS
CD81
Flotillin1
TSG101
CNX
F
Astro sEVs
Particle Count (× 10^14)
Size (nm)
Astro
LPS-Astro
G
2D-Astrocytes
3D-Astrocytes
DAPI
GFAP
Tuj1
Merge
2D
3D
H
DAPI
Tuj1
GFAP
Merge
-LPS
+LPS
I
3D-Astrocyte -conditioned Media
Exo-Spin
3D-Astrocytes
sEVs
J
3D-Astro sEVs
Particle Count (x 10^14)
Size (nm)
K
3D-LPS-activated-Astro sEVs
Particle Count (x 10^14)
Size (nm)

Figure S9

Figure S9. Related to Fig. 7. (A) Immunoblot of sEVs and lysates from cortical neurons. **(B)** NTA of the sEV pellet. **(C)** Purity of primary astrocytes cultures. Enriched cultures of astrocytes from P1-P4 mouse pups were immunostained for the astrocyte marker, GFAP, and the neuronal marker Tuj1. Scale bar, 200 µm. **(D)** LPS activates mouse primary astrocytes. Astrocytes were treated with LPS (1 µg/mL), and RNA was isolated after 6 or 10 days. Relative expression of pro-inflammatory genes was measured by qPCR. **(E)** Immunoblot of lysates and the sEV pellet from primary astrocytes. **(F)** NTA of the sEV pellet. **(G)** Astrocytes are activated upon culturing in 2D plates. Representative immunofluorescence images of astrocytes grown either on a glass surface or in a soft collagen gel. Scale bar, 200 µm. **(H)** LPS activates astrocytes grown in 3D-collagen gels. Astrocytes were treated with LPS (1 µg/mL), fixed after 48 h and subsequently immunostained with GFAP and Tuj1 antibodies. Representative images are shown. Scale bar, 200 µm. **(I)** A schematic representation of sEV purification from astrocytes grown in a soft 3D-collagen gel. CM was collected after 6 days and subsequently sEVs were purified using Exo-spin^{TM} columns. **(J and K)** NTA of the sEVs.

NTA plots are from 1 representative experiment out of 3 independent purifications **(B, F, J, K)**. Values are plotted as the mean ± SEM from 3 independent experiments **(D)**. Statistical significance: *p<0.05, **p<0.01 using two-way ANOVA with Tukey's post-test **(D)**.
